# Measuring particle concentration of multimodal synthetic reference materials and extracellular vesicles with orthogonal techniques: Who is up to the challenge?

**DOI:** 10.1002/jev2.12052

**Published:** 2021-01-12

**Authors:** Robert Vogel, John Savage, Julien Muzard, Giacomo Della Camera, Gabriele Vella, Alice Law, Marianne Marchioni, Dora Mehn, Otmar Geiss, Ben Peacock, Dimitri Aubert, Luigi Calzolai, Fanny Caputo, Adriele Prina‐Mello

**Affiliations:** ^1^ School of Mathematics and Physics The University of Queensland St Lucia Queensland Australia; ^2^ LBCAM Department of Clinical Medicine Trinity Translational Medicine Institute Trinity College Dublin Dublin Ireland; ^3^ IZON Science Ltd., Burnside Christchurch New Zealand; ^4^ Institute of Biochemistry and Cell Biology CNR Via P. Castellino 111 Napoli Italy; ^5^ NanoFCM Co., Ltd, Medicity Nottingham UK; ^6^ European Commission Joint Research Centre (JRC) Ispra Italy; ^7^ Department of Biotechnology and Nanomedicine SINTEF Industry Trondheim Norway; ^8^ AMBER Centre CRANN Institute, Trinity College Dublin Dublin Ireland

**Keywords:** extracellular vesicles, liposomes, multimodal samples, nanomedicine, orthogonal techniques, particle concentration, particle size distribution, polystyrene

## Abstract

The measurement of physicochemical properties of polydisperse complex biological samples, for example, extracellular vesicles, is critical to assess their quality, for example, resulting from their production and isolation methods. The community is gradually becoming aware of the need to combine multiple orthogonal techniques to perform a robust characterization of complex biological samples. Three pillars of critical quality attribute characterization of EVs are sizing, concentration measurement and phenotyping. The repeatable measurement of vesicle concentration is one of the key‐challenges that requires further efforts, in order to obtain comparable results by using different techniques and assure reproducibility. In this study, the performance of measuring the concentration of particles in the size range of 50–300 nm with complementary techniques is thoroughly investigated in a step‐by step approach of incremental complexity. The six applied techniques include multi‐angle dynamic light scattering (MADLS), asymmetric flow field flow fractionation coupled with multi‐angle light scattering (AF4‐MALS), centrifugal liquid sedimentation (CLS), nanoparticle tracking analysis (NTA), tunable resistive pulse sensing (TRPS), and high‐sensitivity nano flow cytometry (nFCM). To achieve comparability, monomodal samples and complex polystyrene mixtures were used as particles of metrological interest, in order to check the suitability of each technique in the size and concentration range of interest, and to develop reliable post‐processing data protocols for the analysis. Subsequent complexity was introduced by testing liposomes as validation of the developed approaches with a known sample of physicochemical properties closer to EVs. Finally, the vesicles in EV containing plasma samples were analysed with all the tested techniques. The results presented here aim to shed some light into the requirements for the complex characterization of biological samples, as this is a critical need for quality assurance by the EV and regulatory community. Such efforts go with the view to contribute to both, set‐up reproducible and reliable characterization protocols, and comply with the Minimal Information for Studies of Extracellular Vesicles (MISEV) requirements.

## 1 INTRODUCTION

Extracellular vesicles (EVs) are small lipid membrane enclosed bodies of size greater than 30 nm in diameter, to be differentiated from intermediate‐density lipoprotein, low‐density lipoprotein, and high‐density lipoprotein that are also present in plasma. EVs can be subclassified into several different subsets based on biogenesis (Akers et al., 2013; Théry et al., 2018), in order of increasing size, such as exosomes (< 150 nm), microvesicles (> 100 nm) and apoptotic bodies (> 100 nm) (Hessvik & Llorente, 2018). EVs have been considered a recent avenue for biomedical investigation in both regenerative and pathogenic contexts, with potential in diagnostic and therapeutic applications (Savage et al., 2018). Their potential use in manifold applications, including drug delivery, particle loading and other theragnostic applications have been reviewed recently (Piffoux et al., 2018).

Physicochemical properties of EVs, such as particle size distribution (PSD), stability, particle concentration (quantification of particle number per unit of volume), and phenotype are critical quality attributes (CQAs) that must be measured in a standardized way to assure quality, potency, stability and batch to batch consistency. Unfortunately the lack of standardized methods for the characterization of those CQAs remains a strong regulatory gap (Lin et al., 2014). There are characterization infrastructures, such as the European Union Nanomedicine Characterisation Laboratory (EUNCL) and the NCI‐Nanotechnology Characterization Laboratory (NCI‐NCL), that endeavour to produce standard operating procedures for the characterization of medically relevant nanoparticles, and that are working for developing regulatory standards in collaboration with regulators and standardization bodies.

Moreover, a set of guidelines for the minimal information required in the study of EVs has been published in 2014 (Lötvall et al., 2014) and updated more recently (Théry et al., 2018). According to those guidelines, different techniques can be used to measure EV physical properties, such as particle size distribution and particle concentration: the most used for EV characterization are single particle analyses such as nanoparticle tracking analysis (NTA) and tunable resistive pulse sensing (TRPS) (Gardiner et al., 2016; Hartjes et al., 2019). A hyphenated technique (that couples a separation with a measurement step), which was recently proposed to measure particle size and PSD is asymmetric flow field flow fractionation, coupled with light scattering detectors (AF4‐MALS or ‐DLS) (Gioria et al., 2018; Naiim et al., 2015; Zhang et al., 2018). Various ensemble techniques might also be able to measure PSD and concentration of polydisperse EV containing plasma samples, including multi‐angle dynamic light scattering (MADLS) and centrifugal liquid sedimentation (CLS) (Langevin et al., 2018; Vegad, 2018). Detection and quantification of nanoparticle mixtures using ensemble (e.g. MADLS, AF4‐MALS, CLS) and single‐particle analysis techniques, (e.g. NTA (Gardiner et al., 2013), TRPS (Vogel et al., 2016) and nano flow cytometry (nFCM)) rely on distinct physical principles.

**NTA** is a light scattering based method that tracks the Brownian motion of each particle individually, in order to determine the mean square displacement of individual particles. With temperature and viscosity of the suspension to be known, the 2D Stokes‐Einstein equation is used to determine hydrodynamic diameters of particles individually. Particle concentration is determined from the number of particles being tracked in an estimated illumination volume (Gardiner et al., 2013). In addition to size and concentration analysis, NTA allows for fluorescence‐based measurements.

**TRPS** is a single particle analysis characterization technique that has been shown to directly measure the PSD, concentration (Roberts et al., 2012; Vogel et al., 2011, 2016; Willmott et al., 2010), and zetapotential (Vogel et al., 2012, 2017) of synthetic and biological nanoparticles, such as EVs. It does this electrically rather than optically, by measuring the change of impedance when an analyte/particle travels through a pore (Maas et al., 2014). Reproducible concentration measurements over a given particle size range are obtained, when using a multi‐pressure calibration procedure, where particle rates for sample and calibration are measured at two or more pressures (Roberts et al., 2012; Willmott et al., 2010). Particle concentrations are calculated from the linear particle rate versus pressure dependence, whilst particle sizes are determined individually from the respective resistive pulse magnitudes. Similarly, to NTA and nFCM, this includes information about the particle concentration distribution (PCD), that is, the concentration of each size population within a sample, given in the number of particles per ml and per size bin (in nm).

**nFCM** is a distinct sub‐set of flow cytometry, benefitting from optical and mechanical adjustments to focus analysis onto < 1000 nm diameter particles, with accurate measurement of EVs down to 40 nm. Single particles are hydrodynamically focused in a sheath fluid stream, and subsequently exposed to laser excitation. Single photon detectors (SPCM) measure both scattered light refracted from these particles as well as fluorescence photons emitted by attached fluorophores (Tian et al., 2018). Regarding to sizing of the EV isolates, intensity of side scattered light is compared to a standard curve generated by measurement of a four‐modal silica nanosphere mixture with a refractive index of approximately 1.45 (Van Der Pol et al., 2014), which is similar to the refractive index of 1.37‐1.42 reported for EVs (Tian et al., 2018, 2020; Van Der Pol et al., 2014; Welsh et al., 2020). High resolution side scatter detection allows for label free detection of all particles (including non‐EV type materials), whilst further fluorescent staining strategies allow for identification of subpopulations (Zhu et al., 2014). The concentration of samples was determined by comparison to 250 nm silica nanoparticles of known particle concentration to calibrate the sample flow rate.

**CLS** measures PSD from the particle sedimentation time in a rotating disc that contains a density gradient of typically sucrose solution. According to Stokes Law, particles are being separated due to differences in size, density and shape (Lamb, 1945). These particles can be subsequently detected, using light based methods (Lamberty et al., 2011; Shard et al., 2018). A particle calibration size standard of known density is typically used, rather than basing measurements on first principles (Linsinger et al., 2012). This method does typically not produce a number‐weighted output of concentration, however it does produce an intensity (extinction) based distribution which can be indicative of the mass‐based concentration distribution present (Linsinger et al., 2012). It has also been shown that the relative concentrations of populations can be mathematically calculated, yet extensive experimental knowledge is required, including the knowledge of particle density and optical properties, and comparison to an independent concentration assessment method may be required to get reliable absolute concentration values (Shard et al., 2018).

**MADLS** is an improved version of single angle dynamic light scattering (DLS), which is an ensemble technique that has been utilized extensively in the field of nanoparticle characterization. It should be noted that the presence of larger particles can obscure smaller particles present and distort the PSD significantly (Hole et al., 2013). MADLS is a promising approach, to produce more accurate PSDs and offer greater size resolution than single angle DLS (Naiim et al., 2015), as populations that may be weakly scattering at one detection angle, are revealed in the other. In MADLS, the PSD is derived from multiple scattering autocorrelation functions recorded at different angles (typically 3), as opposed to a single autocorrelation function in DLS. The determination of total particle concentration involves using measured photon count rates along with material and dispersant optical properties to transform intensity‐weighted PSD into PCD. It is important that the size of the sample at hand is determined as accurately as possible to minimize the propagation of errors in the calculation of total concentration (Technical Note, 2018c).

**AF4‐MALS** couples a size‐based fractionation separation step in an open‐channel (no stationary phase) with an online sizing detector based on static multi‐angle light scattering (MALS). Separation is achieved within a thin flow against which a perpendicular force field is applied. In the normal mode of AF4, particles elute from smallest diameter to largest diameter or from highest to the lowest diffusion coefficient (Wahlund & Giddings, 1987). The hyphenation of AF4 separation coupled to light scattering detectors has demonstrated its power in measuring particle size distribution for a variety of nanoparticle types, polymers, proteins (Mudalige et al., 2018), and was recently proposed as method to measure particle size distribution of EV isolates (Zhang et al., 2018). Despite being mainly used for measuring particle size, AF4‐MALS can also provide the mass‐ and number‐weighted particle concentration, previously tested on virus like particles (Xu et al., 2017). This approach will only work if no sample is lost on the membrane during the fractionation step prior to the measurements (full recovery), and if the optical properties of the samples (e.g. refractive index and absorption) are known.

Each of the techniques described above are deemed sufficient in measuring particle size distribution and concentration in the 50–300 nm range considered in this work, yet it should be taken into account that each platform has specific limitations, for example, a specific range of sizes and concentrations that allow for accurate quantitation, as reported in Table 1. This is in line with International Society for Extracellular Vesicles (ISEV) under Minimal Information for Studies of Extracellular Vesicles (‘MISEV’) guidelines (Théry et al., 2018).

**TABLE 1:** Qualitative and quantitative comparison of six orthogonal methods regarding to their size range, resolution, direct measure of particle concentration, cost and measurement complexity

Parameters	NTA	TRPS	nFCM	CLS	AF4‐MALS	MADLS
Diameter range	30 nm^a^‐600 nm	40 nm‐20 μm (Vogel et al., 2016)	40 nm‐1000 nm	20 nm‐50 μm^a^	20 nm‐500 nm	1 nm‐10 μm
Sample volume	600 μl	40 μl	10‐100 μl	100 μl	20‐100 μl	>50 μl
Cost per sample	moderate	moderate	low	moderate	medium	low
Technical expertise required	medium (Akers et al., 2016; Sitar et al., 2015)	medium (Anderson, et al., 2015)	medium	medium	medium (Oeyen et al., 2018)	low
Time required per measurement	low	low	low	moderate	medium	low
Data analysis complexity	medium	medium	medium	medium	medium	low
Calibration required	yes	yes	yes	yes	no	no
Quality of number‐weighted PSD (see Figure 1, 2, 3, 4)	moderate	high	high	high	medium	low
Number‐weighted concentration measurement?	yes	yes	yes	not direct (light extinction)	not direct (Mie theory)	not direct (autocorrelation)
Resolving Capability (see Figures 2, 3, 4)	medium	very high	very high	very high	high	low
Concentration accuracy of polystyrene measurements (deviation from nominal concentration; as obtained from Table 8)	within 60%^b^	within 10%	within 25%^c^	within 10%	within 10%^b^	within 20%
Concentration reproducibility: averaged CV (as obtained from Table 8)	20^b^	14	15^c^	23	13^b^	36
Data post‐processing for concentration	no	no	no	yes	no	no
Data post‐processing for % distribution	yes	yes	yes	yes	yes	Can't resolve populations within mix
Able to measure EV concentration?	yes	yes	yes	no	no	–

Low < Moderate < Medium < High < Very High.

^a^ sample dependant.

^b^ when outliers of samples G and H for NTA and AF4‐MALS respectively were excluded.

^c^ only 7 of the 10 samples were measured.

As a general guideline, it is necessary to select techniques that are fit for purpose. However, in the characterization of complex biological samples, like EVs, this is particularly challenging since each technique should be able to detect and quantify a very polydisperse distribution of particles in a wide size range. For overcoming this challenge and perform a robust and critical assessment of the whole sample population, it is therefore strongly recommended to combine and compare the results obtained by more than one orthogonal method (Caputo et al., 2019).

Moreover, in order to demonstrate the suitability of each experimental approach to measure particle size and concentration of complex samples, testing reference materials mimicking the complexity of the biological samples, as quality control prior to the measurements, is strongly suggested (Caputo et al., 2019). Unfortunately, a metrology standard reference material that is truly representative of EVs is not yet available for measurements of PSD and concentration. Previously, the best choice to check suitability and experimental performances has been to use polydisperse mixtures of polymeric particles, like polystyrene, despite being very different in their chemical and physical properties, compared with EVs.

An improvement upon this, for optical‐based methods, has been to utilize silica nanoparticles as their refractive index is much closer to the refractive index of EVs. However, their stability over long term storage is less than that of polystyrene. Biological standards have also been suggested for future use such as lyophilized exosome standards, but concerns around their storage, heterogeneity, and robustness currently limit their use (Charoenviriyakul et al., 2019).

The use of NIST‐traceable nanoparticle standards has been adopted as calibration materials for different metrological approaches, with the goal of ensuring instrument fidelity and to offer traceability of measurements (Mansfield et al., 2017). This allows for a robust assessment of the platforms and techniques being utilized to characterize materials of known properties, as well as those of unknown properties, such as EVs. To further support and reinforce the calibration approach with acceptable representative materials, the measurement of a known and stable liposome sample (PEGylated liposomes (Bavli et al., 2019)), was taken as a sequential step to validate the protocols developed with traceable standards. The reasoning behind this is that the liposome sample is more similar in its physicochemical properties to EVs than polystyrene, and possess less stability issues than silica particles. Importantly, it should be noted that no certified particle number concentration standards currently exist, and the development of such concentration reference materials of any kind remains one of the challenges for achieving traceability in concentration measurements (Bavli et al., 2019).

Being aware that each platform has differing strengths and limitations, multiple studies have been conducted to compare various methods for the characterization of nanoparticles generally, and EVs more specifically. Most studies have limited their focus on the study of PSD (Clénet et al., 2018; Lamberty et al., 2011; Langevin et al., 2018; Meli et al., 2012; Nicolet et al., 2016; Varenne et al., 2016), and only a few have compared the capability of multiple techniques to measure total particle concentration. Vestad et al. (2017) reported an inter‐laboratory comparison of size and concentration measurements of EVs and monodisperse polystyrene standards with NTA, emphasizing the effect of instrument optimization of NTA settings on concentration measurements. Grabarek et al. (2019) investigated the capability of microfluidic resistive pulse sensing (MRPS), resonant mass measurement (RMM), DLS and NTA to measure PSDs and concentrations of 300, 495, and 799 nm polystyrene particles (particle number ratio of 2:1:1) individually and as mixture. They also measured liposomes, bacteria and protein aggregates. Van Der Pol et al. (2014) measured PSD and concentration of polystyrene mixtures, EVs and vesicles with transmission electron microscopy, NTA, flow cytometry and TRPS. Recently the work of Mallick et al. has been archived in bioXriv (Mallick et al., 2020). This study compares single‐particle interferometric reflectance imaging sensing (SP‐IRIS), NTA, fluorescence, microfluidic resistive pulse sensing (MRPS) and nanoflow cytometry (nFCM, NanoFCM) for particle concentration measurements. This work, and many others within the EV community are stressing the importance of phenotyping and EV biomarking as additional tools to be taken into consideration for quality control of EV sample source and purity in addition to concentration measurements (Clogston et al., 2020; Savage et al., 2018; Tian et al., 2018; Vogel et al., 2016). It is important to stress that the absence of reproducibility in the quality of EV samples, has too often limited comparability of results in characterization trials. Additionally, the lack of highly repeatable standard protocols for the measurements of all critical sample attributes, combined with the lack of reference materials are also impacting the reproducibility of EV characterization within acceptable industrial confidence variance. Such lack of fundamental knowledge is probably slowing down both the basic research and the regulatory approval process in this area, as reported by some of the authors previous works (Gioria et al., 2018; Hole et al., 2013; Savage et al., 2018).

While attempting to provide clear answers to the EV community in term of reproducibility, accuracy and acceptability, this work is focussed on an in‐depth analysis of the key EV isolate quality attributes: concentration and size distribution. It is our aim to further develop reliable protocols and post‐processing analyses for particle concentration measurements in the 50–300 nm size range.

In this context, we report a detailed and comprehensive comparison of six complementary approaches, including NTA, TRPS, nFCM, CLS, AF4‐MALS, and MADLS, for the determination of concentration and number‐weighted PSDs of heterogenous populations of EV isolates, in a step‐by‐step fashion. First, advanced protocols for the measurement, data analysis, data post‐processing and reporting of number‐based PSD in comparable units are developed for NIST‐traceable polydisperse mixtures. These are made of mono‐ and multimodal (up to quadrimodal) particle populations, ranging from approximately 50–300 nm. Strengths and weaknesses of above nanoparticle characterization techniques are also evaluated. Then we validate the developed approaches by measuring a known liposome sample as an additional EV mimic. Finally, the study is complemented by the measurement of EVs with unknown size and concentration heterogeneity from a complex biofluid, to understand how the analysed methods perform in a realistic scenario. To our knowledge this is the first in‐depth report on method optimization and concentration analysis of a large range of very well‐defined (size, PSD and concentration) polydisperse polystyrene mixtures, liposomes and plasma containing EVs, using six different techniques.

The performances of characterization platforms are compared with an educational purpose to inform researchers in the field and as part of an ongoing process to increase transparency and reproducibility. We hope that this work may help an informed discussion on the respective merits and drawbacks of the different platform platforms for the measurement of complex polydisperse organic nanoparticle samples such as EVs (Van Deun et al., 2017).

## 2 MATERIALS AND METHODS

### 2.1 Polystyrene particles

NIST‐traceable polystyrene particles of 60 nm (+/i‐ 4 nm), 100 nm (+/‐ 3 nm), 152 (+/‐ 5 nm), 203 (+/‐ 5 nm), 240 (+/‐ 5 nm) physical diameters were acquired from Thermo Scientific. These mean diameters were certified by Thermo Scientific using transmission electron microscopy (TEM). Particle concentrations were provided in %(M/V) solids (1% solids in water for all above standards) and respective nominal concentrations in particles/ml were calculated from the mean diameter and the density of polystyrene of 1.05 g/cm^3^ to be 8.4E+13/ml, 1.82E+13/ml, 5.18E+12/ml, 2.17E+12/ml and 1.32E+12/ml for CPN60, CPN100, CPN150, CPN200 and CPN240 respectively.

Due to the lack of traceable information of CPN particle concentrations, the relative nominal concentrations of NIST‐traceable standards, ranging from 60–2500 nm were pre‐analysed with TRPS. As outlined in the Supplementary Info (SI) utmost attention was directed towards the full size and concentration characterization of the NIST‐traceable standards used in this study (see SI/Tables S1&2). Samples, containing mixtures of CPN60, CPN100, CPN150, CPN200 and CPN240 polystyrene beads were then assessed for particle size and concentration using NTA, TRPS, MADLS, CLS, nFCM and AF4‐MALS.

### 2.2 Polystyrene particle mixture preparation

Each of the polystyrene bead standards (CPN60, CPN100, CPN150, CPN200, CPN240) were diluted using D‐PBS (CaCl_2_ and MgCl_2_) + 0.03% Tween‐20. The initial particle concentrations, as calculated by the mean size, % solids and density information, were subject to a volumetric dilution detailed in Table 2 to reduce the concentration of each standard to 10^10^/ml for use in producing the subsequent mixtures.

**TABLE 2:** NIST‐traceable polystyrene particle mode diameters measured with TEM with standard error along with calculated concentration, dilution factor and final concentration

Sample	Mode (nm) by TEM	% Solid (w/V)	Calculated concentration (NPs/ml)	Dilution factor	Final concentration (NPs/ml)
CPN60	60 (+/‐4)	1	8.40*10^13^	8400	10^10^
CPN100	100 (+/‐3)	1	1.82*10^13^	1819	10^10^
CPN150	152 (+/‐5)	1	5.2*10^12^	518	10^10^
CPN200	203 (+/‐5)	1	2.2*10^12^	217	10^10^
CPN240	240 (+/‐5)	1	1.3*10^12^	132	10^10^

Using the set of standards diluted to a concentration of 10^10^/ml, ten mixtures of differing composition were formulated by an independent party. These mixtures (see Table 3), along with the monomodal standards themselves were subject to analysis, using the different techniques, with each operator conducting blind testing to prevent bias.

**TABLE 3:** Number‐based composition of each sample mixture with respective proportions of different NIST‐traceable polystyrene particle sizes, that were measured with various techniques

Code	CPN60	CPN100	CPN150	CPN200	CPN240
A	–	50%	–	50%	–
B	–	–	100%	–	–
C	–	25%	25%	25%	25%
D	–	33.33%	33.33%	33.33%	–
E	–	–	–	100%	–
F	–	100%	–	–	–
G	–	50% (dilute 10x)	–	50% (dilute 10x)	–
H	–	–	–	–	100%
I	–	10%	50%	30%	10%
J	33.33%	33.33%	33.33%	–	–

### 2.3 Liposomes

The PEGylated liposomal formulation used in this study is a research grade equivalent of the drug‐free control of Doxil (liposomal doxorubicin, R) (Bavli et al., 2019). The liposome formulation is made of three different lipids: Cholesterol, HSPC and MPEG‐DSPE, mixed at a weight ratio of 20.7 : 57.9 : 21.9, with a total lipid content of 15.6 mg/ml. Liposomes were purchased from Lipocure Ltd. (Jerusalem, Israel). According to the certificate of analysis, the batch used in this study (batch#500010) is characterized by an average hydrodynamic diameter of 78 nm (z‐ave measured by batch DLS, PdI = 0.04).

### 2.4 Extracellular vesicle (EV) sample preparation

COAG‐NORM lyophilized blood plasma (Diagnostica Stago S.A.S., France) was used for subsequent EV isolation, also defined as ‘small particle isolations’. Plasma samples were prepared, following the manufacturer's instructions. Upon reconstitution, 10 μl of thrombin was added to 1 ml of reconstituted plasma. The blood plasma with thrombin was mixed gently for 15 min. The samples were centrifuged at 3000 × *g* for 15 min. The supernatants were transferred to new microfuge tubes. The centrifugation of the sample supernatants was repeated. The sample was subsequently filtered using a MILLEXGP 0.22 μm filter unit.

The process of EV isolation using size exclusion chromatography (SEC) was conducted using IZON qEV_original/70_ _nm_ size exclusion columns, following the manufacturer's instructions. These columns, provided by IZON Science, contain a resin with a pore size of approximately 70 nm, an optimal separation size of 70–1000 nm, a nominal flow rate of 1.0 ml/min at room temperature, a sample load volume of up to 0.5 ml, a column volume of 10 ml, and a void volume of 3 ml. Columns are pre‐filled with PBS, containing 0.05% sodium azide. The qEV columns isolate EVs in typically less than 15 min, resulting in highly purified vesicles. In the size exclusion process proteins and other contaminating molecules smaller than 70 nm enter the pores of the resin and are delayed in their passage through the column, predominantly eluting beyond the EV‐containing volume. The majority of EVs mainly elute in 1.5 ml after the void volume of 3 ml** **[1,2]. A total of 500 μl samples were subsequently collected and aliquot samples provided for measurements to all partners.

### 2.5 Nanoparticle tracking analysis

Sample dilutions for NTA were made up, using D‐PBS (CaCl_2_ and MgCl_2_) + 0.03% Tween‐20 to a working volume of 1 ml, in order to obtain an optimum particle concentration for NTA (between 10 and 50 particles per field of view). Samples were vortexed briefly before loading to ensure adequate mixing and homogeneity. The samples (∼600 μl) were then loaded into the NanoSight instrument.

NTA‐analyses of the NIST polystyrene particles, dilution buffer, liposome sample and the EV containing plasma sample were conducted using the NS500 NanoSight (Malvern Panalytical, UK) along with the Nanosight 3.2 software package (NTA build 3.2.16) following the European Union Nanomedicine Characterisation Laboratory (EUNCL) approved protocol (Maguire et al., 2018). A 405 nm laser was used to visualize particles, present in a given field of view. A total of 60 s recordings of the laser interacting with particles are captured using an EM‐CCD camera. The camera level and focus were manually controlled and chosen by the operator. The detection level was optimized by the operator and the recordings were subsequently analysed by the Nanosight 3.2 software, to determine particle numbers per frame and sample concentrations. Through the phenomenon of Brownian motion, the particle size can be determined by the software. The D‐PBS + 0.03% Tween‐20 used in the dilution of the standards and samples was also analysed to assess background particle levels. Polystyrene latex microspheres (Malvern Panalytical, UK) of known size (100 nm) were used as calibration for NTA measurements.

All samples (polystyrene, liposome and EV isolates) were measured in triplicate. Measured number‐weighted distributions for all samples were averaged over repeat runs and plotted in histogram format with a bin‐size of 5 nm. The concentration of the EV isolates in a sample was reported in two different pre‐defined size ranges of 80–250 nm and 50–400 nm respectively, in order to make concentration results comparable between techniques.

For most multimodal polystyrene samples PSDs of the various modes within the mix overlapped and hence postprocessing of the PSDs was required to determine %distribution of various particle sizes in the mix. For this purpose, multi‐peak Gaussian fitting was applied, using Origin lab 9 software. Both the peak position and the width of the Gaussians were typically independent variables unless otherwise stated.

NTA was conducted for each of the NIST‐traceable standards prior to analysis of the mixtures to give a preliminary estimation of the mode particle diameters of the monomodal standards. Once this was completed, the 10 different mixtures, each designated with a code to preserve blind testing were given to the NTA operator for measurement, which was also the case for all other techniques. The codes assigned to each mixture and their composition are detailed in Table 3.

In the process of adding nFCM to the instrumental techniques used for this comparison study the polystyrene mono‐ and multimodal samples were evaluated again with NTA and TRPS and results compared with the original measurements and results. NTA instrumental settings were kept the same to make measurements comparable. This was done to confirm the stability and integrity of the polystyrene based samples from the time of the original measurements until the nFCM measurements were completed.

### 2.6 Tunable resistive pulse sensing

TRPS belongs to the family of resistive pulse sensing analytical techniques, in which colloidal particles, suspended in a conductive solution, pass through a single or multiple pores in a membrane and produce resistive pulses. These electrical pulses can be collected and analysed by the IZON Science Control Suite v. 3.3, which acts as the interface to qNano and performs calibration required to convert number and magnitude of measured pore blockades to particle concentration and diameter respectively.

PBS containing surfactant (0.03% Tween‐20) was placed in both fluid cells that contain one electrode each, below (75 μL) and above (35 μl) the nanopore on the qNano. Its analogue digital converter operates at 1 MHz, reduced through electronic filtering to a sampling rate of 50 kHz. The system was equipped with an air‐based pressure module to apply the required pressure range. Tunable nanopores were fabricated in TPU membranes (Elastollan1160D, BASF), as detailed in (Sowerby et al., 2007; Willmott et al., 2010).

Size and concentration calibrations were performed using CPN100 and CPN200 standards at a concentration of 10^10^/ml. Multimodal CPN (sample A‐J), liposome and EV isolate concentrations were determined using a two point and three point pressure method (Roberts et al., 2012) respectively, with pressures typically ranging between 0.3–2 kPa and typical sample volumes being 35‐40 μl. Only sample G with the lowest nominal concentration (10^9^/ml) was recorded with a one‐point method at a high pressure, due to low counts. The multi‐point pressure method eliminates the impact of pore and particle zeta potentials (electrokinetic effects) on the detected concentration.

All polystyrene (A‐J), liposome and EV isolate samples were run in triplicate. Measured number‐weighted distributions for all samples were averaged over repeat runs and plotted in histogram format with a bin‐size of 5 nm. The ten separate polystyrene particle mixtures, each designated with a code to preserve blind testing were given to the TRPS operator for measurement. The calibration measurement in all cases preceded the sample measurement at the same applied pressure. For the polystyrene sample, one calibration measurement was done for all three replicate sample measurements, whilst for the liposome and plasma samples, calibration and sample were measured in alternation (3 x calibrations and 3 x samples). The alternating calibration process virtually eliminates the impact of any change in pore geometry occurring during the measurement process on liposome and EV isolate size and concentration results and hence guarantees the most reliable results.

EV isolates were measured using a standardized methodology (Vogel et al., 2016) which is based on a combination of the EV isolation with size exclusion columns (qEV) and their consecutive measurement with TRPS. The methodology also includes the reporting of EV isolate concentration over a defined size range and coating the pore with IZON coating solution. IZON coating solution (ICS, as part of IZON reagent kit, IZON Science, New Zealand) was used to coat the thermoplastic polyurethane nanopores (NP150) before measuring the EV isolates. Coating solution reduces non‐specific binding (NSB) onto the nanopore surface and hence increases the stability of TRPS measurements of EV isolates. The concentration of the EV isolates in a sample was reported in two different pre‐defined size ranges of 80 nm ‐ 250 nm and 50 nm – 400 nm respectively. Blockade counts pertinent for these studies typically were 1000 events/run.

TRPS analysis (PSD and concentration) requires no data post‐processing. For samples C and D, there were small amounts of particles with diameters between 50 and 80 nm (< 3% of total concentration, possibly due to contaminants). For the purpose of measuring %distribution of CPN100 in multimodal mixes, these particulates were excluded from the analysis, but they were added to the total concentration.

### 2.7 Nano flow cytometry

Nano flow cytometry uses specialized equipment to apply the fundamentals of standard flow cytometry to sub‐micron particles. The NanoAnalyzer (nanoFCM) has been optimized to allow for side scatter (SSC) measurements of biological particles, such as EVs and viruses, down to 40 nm, in line with the comparative study of Ma et al. (2016). Two additional fluorescent detectors allow for further characterization of particles (Zhu et al., 2014).

A NanoAnalyzer N30 instrument equipped with a single 488 nm laser and single‐photon counting avalanche photodiodes detectors (SPCM APDs) was used for detection of the SSC (bandpass filter: FF01 − 524/24) of individual particles/EVs. HPLC grade water served as the sheath fluid via gravity feed, reducing the sample fluid diameter to ∼1.4 μm. Data were generated through the NanoFCM Professional Suite v1.8 software, with noise being removed through the use of blanks.

Measurements were taken over 1‐min periods at a sampling pressure of 1.0 kPa, modulated and maintained by an air‐based pressure module. Samples were diluted in PBS as required to allow for 2000‐12000 counts to be recorded during this time.

During data acquisition the sample stream is completely illuminated within the central region of the focused laser beam, resulting in approximately 100% detection efficiency, which leads to accurate particle concentration measurement via single particle enumeration (Tian et al., 2020; Zhu et al., 2010).

The concentration of samples was determined by comparison to 250 nm silica nanoparticles of known particle concentration to calibrate the sample flow rate. Sizing calibrations were specific to certain subsets of samples as were laser settings. EV isolate and liposome samples were sized according to standard operating procedures using the proprietary four‐modal silica nanosphere cocktail generated by nFCM to contain nanosphere populations of 68 nm, 91 nm, 113 nm, and 155 nm in diameter. A standard curve is produced by fitting the side scatter intensity vs particle diameter of the four different silica particles with a power function f = c*d^n^. For the EV and liposome measurements the best fit for the silica cocktail was achieved with an exponent n of approximately 5.3. The produced standard curve is then used to size liposomes and EVs. Please note that in case of Rayleigh approximation that typically applies for particle diameters d smaller than 1/10 of the wavelength of the incident light – in our case of the laser wavelength being 488 nm Rayleigh theory applies for d < 50 nm – the scatter intensity scales with d^6^.

Silica provides a stable and monodisperse standard with a refractive index of approximately 1.43‐1.46 (Hart & Terray, 2003; Van Der Pol et al., 2014; Welsh et al., 2020) which is close to the range of refractive indices reported in literature for EVs (n = 1.37‐1.42) (Tian et al., 2018, 2020; Van Der Pol et al., 2014; Welsh et al., 2020). Using such a calibration standard enables accurate nFCM size measurements, as confirmed when comparing nFCM with cryo‐TEM results (Tian et al., 2018). The refractive index of liposomes is also assumed to be similar to silica, with variations accrued by their lipid and biological composition, remaining important considerations during data interpretation (Matsuzaki et al., 2000). The laser was set to 10 mW and 10% SSC decay.

As polystyrene has a very different refractive index compared with silica, polystyrene samples were sized by comparison to multimodal cocktails of CPN and NIST particles. Side scatter intensities measured for particles in mixed samples (C, I, J) were compared to a trimodal cocktail of CPN 60, CPN100, CPN150. These measurements were taken at a laser power of 25 mW, 0.2% SSC decay, allowing for inclusion of all particles in a single 1‐min measurement.

Data processing was handled within the nFCM Professional Suite v1.8 software, with dot plots, histograms, and statistical data being provided in a single PDF. Gating within the software allows for proportional analysis of subpopulations separated by SSC intensities with PSD and concentrations available for each sub‐population. In cases where additional contaminant particles were observed (past the frequency observed in the blanks) thresholding was applied to remove these from further processing. Conversion of PSD histograms to show particles/ml on the y axis was done by exporting data as CVS files to allow for Excel data conversion. Briefly, histogram data were exported, and number of events was converted from integers to particles per ml using the concentration standard measured.

### 2.8 Centrifugal liquid sedimentation

Centrifugal liquid sedimentation, using a CPS Disc Centrifuge (model DC24000 UHR, CPS Instruments, Inc., USA) separates particles by size using centrifugal sedimentation in a liquid density gradient medium. The sedimentation process leads to variations in the particle concentration in front of the detector when measured in time. Sedimentation times correspond to the settling velocities, which in case of isolated particles depend on their individual size, shape, and density. The instrument applies the Stokes law to calculate the size that corresponds to a certain sedimentation time at a given density. Mie theory is applied to calculate the mass concentration of particles from the measured light extinction using the material specific refractive index and absorption values at the wavelength of the light source (405 nm). Mass‐weighted size distribution curves are generated by the instrument's software considering the geometric features of the disc and the position of the detector.

For the separation, a density gradient made of 9 steps of 1.6 ml of 0%‐8% sucrose solution was used at a rotation speed of 22000 rpm. Refractive indices of 1.62 and 1.42, and densities of 1.05 g/ml and 1.07 g/ml were applied in the calculations for polystyrene and EV respectively. The absorption for both polystyrene and EV isolates was set to 0.001. To protect the sucrose gradient against water evaporation, 0.5 ml of gradient cap fluid (dodecane, CPS Instruments, Inc.) was added on top of the last layer. A CPS size calibration standard (lot. 150, CPS Instruments, Inc.), that is, an aqueous suspension of monodisperse spherical polyvinyl chloride (PVC) particles with a diameter of 237 nm, was injected before each individual measurement, in order to determine the actual properties (density, viscosity) of the gradient.

In our experiments, the mass of the syringe loaded with about 100 μl suspension before injection and the mass of the syringe after injection were measured using an analytical balance. Supposing that the nanoparticles at the applied concentration have negligible effect on the effective density of the suspension, the mass measurement allows the calculation of the injected volume and thus the mass concentration from the mass‐weighted distribution data. Particle concentration distributions (PCDs; particles/nm/ml) were transformed from mass‐weighted PSDs (ng/nm), dividing the particle diameter dependant total mass by the respective single particle mass and injected volume. The particle mass is the product of particle density (1.05 g/ml for polystyrene) and particle volume supposing that the particles have spherical shape.

The 10 separate polystyrene mixtures, each designated with a code to preserve blind testing, were given the CLS operator for measurement. In order to obtain reliable and reproducible PCDs and total concentrations for the mono‐ and multimodal polystyrene mixes mass‐weighted PSDs require data post‐processing. Although CLS was baseline‐corrected by the software, most mass‐weighted PSDs were manually baseline‐subtracted post‐processing using Origin Lab 9, before converting them into respective number‐weighted PSDs. This manual post‐analysis correction was necessary because measured mass‐weighted PSDs show a significant drift to either negative or positive values in particular for particle diameters smaller than 100 nm. Since for an equal mass of small and large particles the smaller ones are overrepresented in number, this drift can significantly distort calculated PCDs. Unfortunately, in most cases the composition of the particles within a mix need to be well known, in order to perform a correct baseline‐correction, particularly for the smaller particles within the mix.

After the data post‐processing, total particle concentrations and %distributions of various modes within a mix were calculated, using Origin Lab 9. For the calculation of the %distributions of various polystyrene particle populations within the mix the peaks of PCDs were integrated over known particle size ranges. If the particle size range of the particles at hand is not known the integration might include artefacts, interpreted as particles with small diameter and significantly distort PCDs and total concentrations. Integration intervals were chosen from the onset of the first particle‐based peak to the midway positions between peaks, to the end of the largest peak (= largest particle population within mix).

For EV isolate measurements, its concentration was reported in two different pre‐defined size ranges of 80–250 nm and 50–400 nm, respectively.

### 2.9 Asymmetric flow field flow fractionation online with a multi‐angle light scattering detector (AF4‐MALS)

The AF4‐system used in this study included an Eclipse Dualtec separation system (Wyatt Technology Corp., Santa Barbara, CA, USA) and an Agilent 1260 Infinity high‐performance liquid chromatograph equipped with a degasser (G1322A), an isocratic pump (G1310B), an autosampler (G1329B) and a multi‐wavelength detector (G1365C), all from Agilent Technologies (Agilent Technologies, Santa Clara, USA). A DAWN 8+ HELEOS II multi‐angle laser light scattering (MALS) detector operating with a 658‐nm laser (Wyatt Technology) was coupled to the fractionation system. The 90° detector angle was used to monitor the signal during analysis. Regenerated cellulose (10 kDa) membranes were used in the Eclipse SC separation channel (153 mm length). The spacer height was 350 μm. The temperature of the channel was kept constant at 25°C.

Three different eluents and flow programmes were used for polystyrene, liposomes and EV isolate samples respectively. For the analysis of the polystyrene samples, the eluent was 0.05% sodium dodecyl sulfate in ultrapure water. The flow programme and cross‐flow settings are included in Table 4. The detector flow was set at 1.0 ml/min, the injection volume was 50 μl and the focus flow was 0.8 ml/min. For the analysis of liposomes all settings were taken from (Parot et al., 2020), while for the analysis of EV isolates all settings were taken from a recently published methodology (Zhang & Lyden, 2019), with the exception of the channel height, using a 350 μm spacer instead of the recommended 490 μm spacer. In the case of the EV isolates, unfortunately the analysis of the particle size and concentration described above was not successful, due to the low light scattering signal and hence low signal/noise ratio, which did not allow to perform reliable size analysis at the actual sample concentration.

**TABLE 4:** AF4 flow programme and cross‐flow settings for the measurement of polystyrene particle mixtures

Time [min]	Step	Cross‐Flow Start [ml/min]	Cross‐Flow End [ml/min]
0 ‐ 2	Elution	0.8	0.8
2 ‐ 4	Focus		
4 ‐ 9	Focus + Inject		
9 ‐ 16	Focus		
16 ‐ 21	Elution	0.8	0.5
21 ‐ 26	Elution	0.5	0.4
26 ‐ 36	Elution	0.4	0.3
36 ‐ 76	Elution	0.3	0.1
76 ‐ 77	Elution	0.1	0
77 ‐ 83	Elution	0	0

The data acquired with the MALS detector for polystyrene mixes and liposomes were processed using the ASTRA 6.1 software package (Wyatt Technology Corp., Santa Barbara, CA, USA). For sizing measurements of polystyrene particles the Lorentz‐Mie model was applied, while for liposomes the sphere model was used. In both cases, the gyration radius (Rg) is determined.

PSD and PCD were determined by applying the ‘number density’ method, assuming a refractive index of 1.59 and of 1.392 for polystyrene and liposomes respectively. The refractive index of the liposomes was selected according to (Matsuzaki et al., 2000) and is derived as the volume‐square‐average refractive index of the lipids (1.45) and water core of the liposome (1.331) assuming an average diameter of 75 nm and mono‐lamellar particles. Please note that a small variation of the refractive index that may derive from a change in liposome size or from the presence of multilamellar particles will impact the particle concentration measurements derived by light scattering data, and thus care should be taken to use a realistic average refractive value, or to measure it prior to AF4‐MALS analysis.

The particle size distribution calculated by the ‘number density’ method is currently only reported by the software in arbitrary units. However, thanks to a collaboration with Wyatt Technology we developed an algorithm to transform the particle size distribution reported by the software in arbitrary units into a number‐based particle size distribution in particles/nm/ml. The excel macro is available for the readers as Supplementary Info #2 and detailed instruction for its use are provided in the main Supplementary Info. The procedure used here is expected to be implemented in a later version of the ASTRA software. Importantly, the authors and the instrument provider (Wyatt Technology Corp., Santa Barbara, CA, USA) discourage the use of the differential number or differential mass‐based size distributions provided by the ‘particles’ method, which are not accurate for particle concentration measurements, and suggest to refer to the ‘number density’ method for concentration measurements.

The following parameters derived by AF4‐MALS were reported:

- Complete fractogram(s) of the eluted sample, showing the elution time on the x‐axis and the detector response(s) (UV‐VIS and diameter of gyration = Dg) on the y‐axis (SI/Figure S3).
- The mode value of the diameter of gyration (value at the peak maximum of the UV‐VIS concentration detector).
- Number‐based particle size distribution in particles/nm/ml
- The total number‐based particle concentration (particles/ml) derived by integrating number‐based PSD.
- The number% of the relative concentration of the different particle populations, when peaks of different populations were resolved.

### 2.10 Multi‐angle dynamic light scattering

MADLS was conducted using the Zetasizer Ultra (Malvern Panalytical, UK). Typical sample volumes were approximately 1 ml, loaded into a DTS0012 cuvette. Each sample's size distribution, mode particle size and particle concentration were measured using detectors at three different angles to account for front (13°), side (90°) and back (NIBS 173°) scatter of light. Each sample was measured at each angle in triplicate with adaptive correlation applied to each to improve overall data quality. Data were processed using the ZS Xplorer Software Suite V 1.1.0.656.

The Zetasizer Ultra analysis includes total concentration, and both intensity‐ and volume‐ weighted PSDs. The volume‐weighted PSDs were post‐processed in order to produce respective concentration distributions. Data post‐processing included the conversion of volume‐ into number‐weighted PSDs and integrating the converted PSDs. Finally, the height of the number‐weighted PSDs was adjusted, so respective PSD integrals became equal to the total particle concentrations.

Before the actual measurements the MALDS instrument was calibrated for size measurements using a mix of 200 nm and 400 nm polystyrene latex microspheres (Malvern Panalytical, UK). MADLS was conducted for 4 of the 5 (besides CPN60) NIST‐traceable standards (Table 2) prior to analysis of the mixtures to give a preliminary estimation of the mode particle diameters of the monomodal standards. Once this was completed, nine separate polystyrene mixtures, each designated with a code to preserve blind testing, were given to the MADLS operator for measurement.

### 2.11 Statistical analysis

GraphPad Prism version 7.00 for Windows (GraphPad Software, La Jolla, California, USA, www.graphpad.com) was used to determine the mean and mode particle diameters and total particle concentrations along with the standard error and coefficient of variance of the mean for each sample measured with NTA. D'Agostino‐Pearson omnibus normality test and Shapiro‐Wilk normality test were used to assess the normal distribution of data acquired. Average values and SEM were acquired using column statistics and 1‐Way ANOVA followed by Tukey's multiple comparison tests with a single pooled variance. Statistical significance was set at *P* < 0.05.

The goodness‐of‐fit of multimodal Gaussian fit of NTA PSDs for polystyrene mixes was quantified by calculating the coefficient of determination (R^2^), using Origin 9.

Coefficient of variance (CV = standard deviation/mean) for repeat measurements of total particle concentrations were calculated for all samples and different techniques. Accuracy for size and concentration measurements were quantified by calculating the measurement bias in %, which is defined by the difference of nominal and measured value, divided by the nominal value and multiplied by 100.

### 2.12 Comparison of concentration units reported by the different techniques

Whilst NTA, nFCM and TRPS directly measure size distributions in particles/ml, CLS and AF4‐MALS measurements are initially intensity‐weighted but can be converted with the knowledge of particle density and refractive index into number‐weighted PCDs in particles/nm/ml. CLS crucially requires post‐processing of the data, otherwise total concentrations of smaller particles can be overestimated. For this reason, CLS measurements were baseline‐subtracted where required post‐processing. MADLS PSD measurements are provided in both an intensity and volume weighted format. Volume weighted PSDs were then converted into number‐weighted PSDs in a post‐processing step.

## 3 RESULTS AND DISCUSSION

In this study we have performed measurements in four steps of incremental complexity. First, we have measured the size and concentration of monodisperse NIST‐traceable standards ranging from 50–300 nm. In a second step, we have prepared complex multimodal NIST‐traceable polystyrene standards, in order to mimic the size distribution of typical EV containing plasma samples. Third, we validated all the protocols developed by measuring a PEGylated liposome sample. Finally, we have measured PSD and concentration of one EV containing plasma sample. In all steps the performance of six techniques were compared, including NTA, TRPS, nFCM, CLS, AF4‐MALS and MADLS. As stated in the introduction, the benefit of using multimodal NIST‐traceable samples as EV mimics in step 1 and step 2 is that their PSDs and total concentrations are well known. Hence performance of various platforms can be evaluated in a quantifiable way, while liposomes are chosen in step 3 as the synthetic lipid‐based vesicles closer in nature to EVs. Lastly, step 4 demonstrates the complex particle size distribution and concentration measurements of EVs extracted from an established single isolation process using commercial SEC.

### 3.1 NIST‐traceable standard mixtures

The composition of the NIST‐traceable standard mixtures used for analysis by each technique are detailed below (Table 3). The nominal concentration for 9 of 10 polystyrene samples was 10^10^/ml, sample G, that was diluted 10x had a final concentration of 10^9^/ml. Utmost attention was directed towards the full characterization of the NIST‐traceable standards used in this study and concentrations were evaluated carefully prior to measurements as detailed in SI (SI/Tables S1 and S2). Whilst mean diameters of various standards were determined by the particle provider with TEM, concentrations as derived from the %solid information were pre‐measured with TRPS and showed to be in very good agreement with nominal concentrations (Table S2).

Measurements taken with the faster techniques, such as NTA, TRPS, nFCM, and MADLS were taken in triplicate, while measurements with CLS and AF4‐MALS that require a lengthy setup were taken in doublet or in singlet, respectively.

### 3.2 Measurement of monomodal polystyrene samples

**FIGURE 1:**
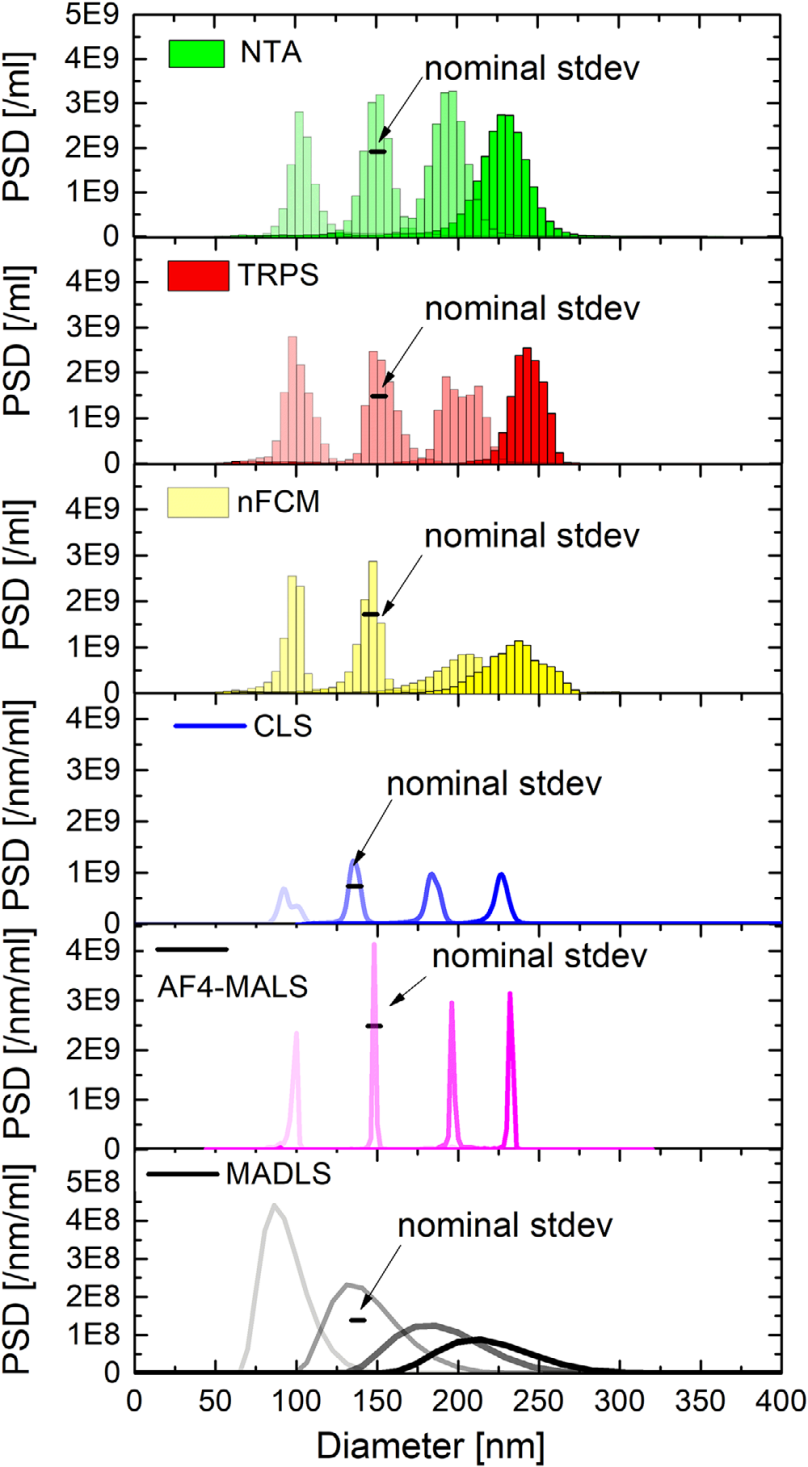
NTA, TRPS, nFCM, CLS, AF4‐MALS and MADLS measurements of monomodal CPN100, CPN150, CPN200 and CPN240. The nominal standard deviation of the NIST‐traceable CPN150 standards as given by the particle provider are indicated for all six techniques. NTA, TRPS, nFCM and MADLS measurements were averaged over 3 runs and CLS over 2. AF4‐MALS was only performed once. AF4‐MALS measurement of sample H was scaled down by a factor of 5, to make it comparable with the other sample measurements

Number‐weighted size distributions of the monomodal samples (B, E, F and H) are shown in Figure 1 and mode diameters and total concentrations are listed in Table 5. For reasons of simplicity and consistency we state mode diameters as opposed to mean diameters. For near‐Gaussian distributions as in case of NIST‐traceable CPN100, CPN150, CPN200 and CPN240 the discrepancy between mean and mode diameters will be typically below 3%.

**TABLE 5:** Total measured concentrations, CVs and mode diameters for monomodal polystyrene samples F (CPN100), B (CPN150), E (CPN200) and H (CPN240)

Technique	Mean concentration of F/B/E/H (NPs/ml)^*^10^10^				Mean concentration CV (%) of F/B/E/H				Diameter (nm) of F/B/E/H 100/152/203/240			
NTA	1.10	1.39	1.85	2.06	31.3	7.6	19.6	22.9	103	153	195	229
TRPS	1.06	1.10	1.15	1.33	8.3	2.1	13.3	4.6	98	148	203	243
nFCM	0.79	0.94	0.72	0.96	7.5	3.7	12.4	11.1	98	147	207	238
CLS	0.77^a^	1.14	1.08^a^	1.11	21.1	4.4	17.5	7.2	97^b^	136^b^	185	227
AF4‐MALS	1.07	1.01	0.97	5.80	–	101	148	196	232			
MADLS	1.43	1.20	0.85	0.62	0.6	2.1	3.9	7.8	106	151	200	230
									*87* ^c^	*133* ^c^	*182* ^c^	*214* ^c^

^a^ without baseline‐subtraction total concentration can be much higher; for example, for sample E and F max concentrations can be up to 50x higher than baseline‐corrected concentrations (see SI/Table S3).

^b^ run 2 for sample B has a satellite peak at 169 nm (< 3%) and run 1 for sample F additional peaks at 180 and 313 nm (< 1%).

^c^ as derived from PCD (slanted) as opposed to intensity‐weighted PSD.

Mode diameters of the mono‐modal distributions as measured with NTA, TRPS, nFCM, AF4‐MALS and MADLS are in very good agreement (within 6%) with nominal diameters for all samples. Only CLS shows consistently smaller particle diameters (up to 11%), with a similar shift being observed in a previous comparison study (Anderson et al., 2013). Please note that NTA, MADLS, and nFCM all measure the hydrodynamic diameter, CLS measures Stokes diameter, AF4‐MALS the radius of gyration and TRPS measures the geometric particle diameter (see appendix/glossary). Whilst hydrodynamic and geometric diameters of polystyrene particles can be quite different, they become comparable at higher salt molarities (Sasaki, 1984), as in case of PBS, that was chosen as dispersant, and hence become comparable.

Averaged number‐weighted PSDs obtained from the unmixed monomodal analysis of CPN100, CPN150, CPN200 and CPN240 particle samples are shown in Figure 1, and the numerical values reported in Table 5. It was not possible to present the number‐weighted PSDs in the same format for all techniques due to the different ways these six dissimilar techniques measure and calculate PSDs. NTA, TRPS and nFCM size distributions are displayed in histogram format (with 5 nm bin size) as typically done for particle by particle measurement techniques and were averaged over 3 runs each. CLS, AF4 and MADLS are presented as continuous measurements of detected particles with no data binning as these platforms are based on ensemble and averaging operating principles, with results being averaged over 2, 1 and 3 repeat runs respectively.

The averaging of the size distributions over 3 (for NTA, TRPS, nFCM and MADLS) and 2 runs (CLS) leads to a slight broadening of the distributions compared with single runs. In case of the CLS runs of the CPN100 this leads to a double peak distribution due to the size shift between the two repeat runs. AF4‐MALS data are reported for the first time in PSDs by particle number/ml as opposed to arbitrary units, thanks to a custom‐made macro, that was designed during this work jointly with Wyatt Technology for the purpose of obtaining particle concentration measurements outcomes comparable with other techniques (see materials and methods section & SI for more technical details).

In terms of the widths of the size distributions, NTA, TRPS, nFCM and CLS are within the expected nominal distribution widths (standard deviation), whilst AF4‐MALS number‐weighted size distributions are too narrow, and MADLS distributions too broad. The NIST‐traceable standards come with specified mean diameters, standard deviations and respective coefficient of variances (CVs) for respective size distributions, as measured with transmission electron microscopy. Nominal distribution standard deviations are 7.8 nm (CV = 7.8%), 5.0 nm (3.3%), 5.3 nm (2.6%), and 3.7 nm (1.5%) for CPN100, CPN150, CPN200, and CPN240 respectively. CPN150 distributions were used to demonstrate the degree of agreement between measured and nominal size distribution widths for different techniques. For the CPN150s the length of the black bar in Figure 1 indicates twice the nominal standard deviation of the distribution as given by the particle provider. Please note that for a normal distribution its doubled standard deviation represents the distribution width at approximately 60% of its maximum height.

In terms of total concentrations (Table 5), when comparing single‐particle techniques NTA, TRPS and nFCM, NTA measures statistically significantly higher total concentrations (particular for CPN200 and CPN240), if compared with nominal concentrations (10^10^ particle/ml). The opposite trend has been observed for MADLS, underestimating the particle concentration of large particles. Please note, that the validity of these trends would have to be verified with many more measurement repeats. An anomaly was observed in the AF4‐MALS measurement of sample H (CPN240). Whilst CPN100, CPN150 and CPN200 concentrations, as measured with AF4‐MALS were in perfect agreement (within 7%) with the nominal concentration of 10^10^/ml, the measured concentration of CPN240 was more than 5x higher. This was most probably due to a dilution error. For this reason, the PSD of the CPN240 was scaled down by a factor of 5, to make it comparable with the other sample measurements. CVs in Tables 5, 6, 7, 8 are related to the variation in measured concentration of respective repeat runs, three for NTA, TRPS, nFCM and MALDS and two for CLS.

Recorded CLS data for the monomodal samples in some cases crucially relies on data post‐processing, that is, baseline‐subtraction (see experimental section + SI/Figure S1) and the exact knowledge of the composition of the sample (SI/Figure S2), in order to set the correct size intervals for calculation of particle concentration. When reaching the lower size and concentration limits of CLS, the primary data becomes very noisy and the subsequent mass‐ and number‐weighted PSDs can show artefacts. These artefacts are possibly introduced by the transformation of instrument noise to signal during calculations. This is exemplified in Figure S2, that displays mass and number‐weighted PSDs of samples E (CPN200) and F (CPN100). In both cases there appears to be particles with a mean diameter of 60 nm which are not present in the actual sample. In these two cases the total measured concentration increases by up to 100% when including the artefact population. The importance of setting a baseline, in particular for diluted samples, is illustrated in Figure S1. Measured maximum concentrations without baseline‐subtraction can be up to 50x higher (see sample G, SI/Figure S1). This illustrates quite clearly the need for using CLS inside its limits of applicability, that in the case of polystyrene nanoparticles should not go below 80 nm at the actually applied particle concentrations.

### 3.3 Measurement of multimodal polystyrene compositions

#### 3.3.1 Bimodal and trimodal mixtures

In a second step, we tested the capability of the different techniques to measure the concentration and resolve populations characterized by different sizes, increasing the complexity gradually, starting with bimodal and trimodal polystyrene mixtures. Whilst there is a high performance and fair agreement of all tested techniques for monomodal standards in terms of size, size‐distribution and concentration (as shown in the previous section), discrepancies between the different techniques are detected when mixtures are measured, mainly related to the differing capability between techniques of resolving various populations within a polydisperse mix.

**FIGURE 2:**
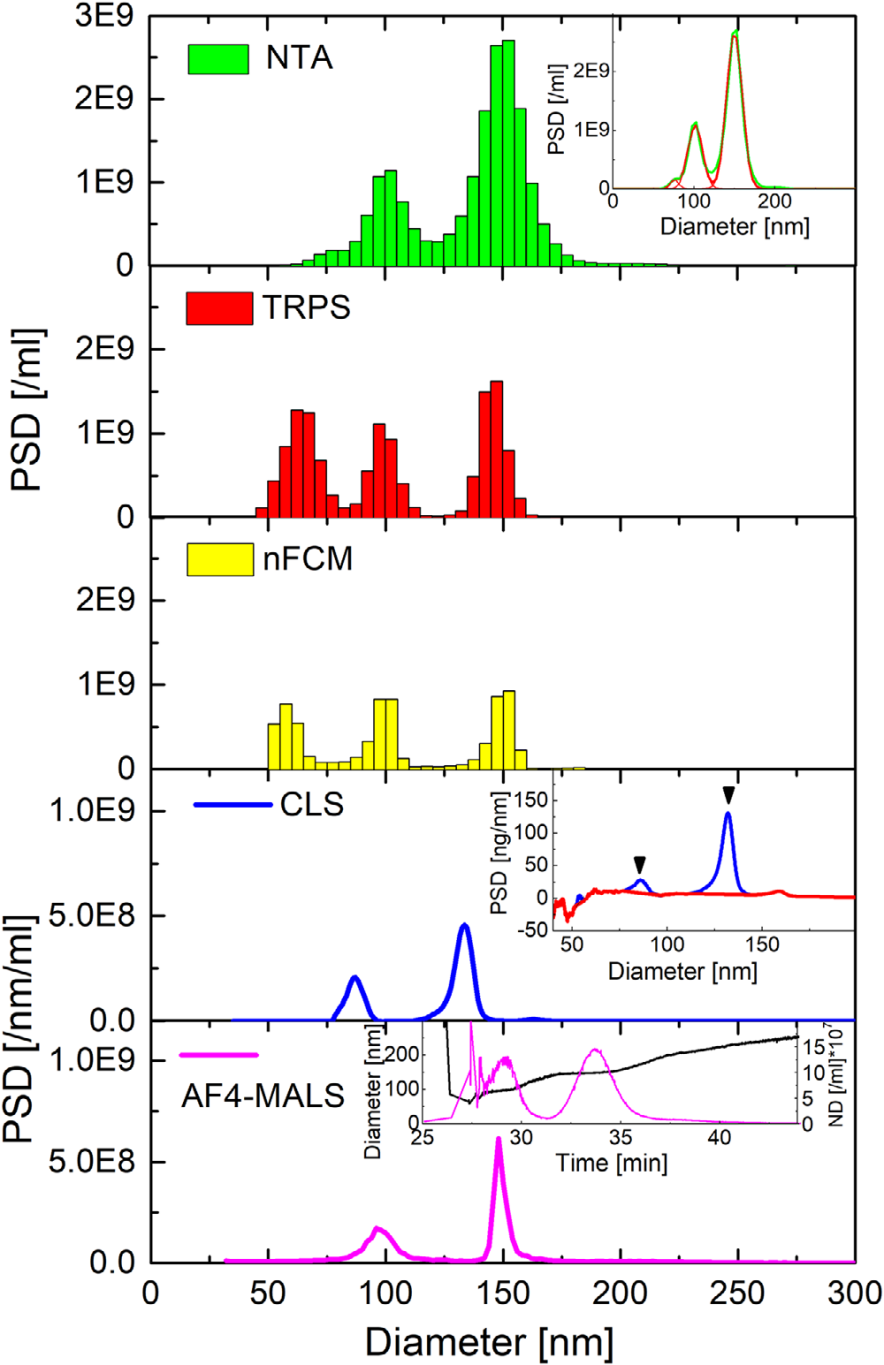
NTA, TRPS, CLS, nFCM and AF4‐MALS measurements of trimodal sample J (CPN60/CPN100/CPN150 at 1/1/1). NTA, TRPS and nFCM measurements were averaged over 3 runs, and CLS over two runs. AF4‐MALS was only performed once. The NTA inset shows the multi‐Gaussian fit of its PSD. The CLS inset shows the baseline‐correction of the mass‐weighted PSD, whilst the AF4‐MALS inset shows the respective number density‐based fractogram and sample specific particle peak positions

When a bimodal mixture of CPN100 and CPN200 (samples A and G; SI/Figure S4 &Table S4) is tested, all methods but MADLS can resolve the bimodal population very well. When incrementing the sample complexity to trimodal mixtures, such as samples D (CPN100+CPN150+CPN200; SI/Figure S5 & Table S5) and J (CPN60+CPN100+CPN150; Figure 2, Table 6) multiple techniques partially fail resolving the different populations in the samples.

**TABLE 6:** Measured mean concentrations (including CVs), mode diameters for various populations within the mix and respective % distributions for trimodal sample J (CPN60/CPN100/CPN150 at 1/1/1)

Technique	Mean concentration (NPs/ml) *10^10^	CV (%)	Diameter (nm)60/100/152			% Distribution1/1/1		
NTA	1.88	4.8	76^a^	101^a^	150^a^	2	27	71^a^
TRPS	1.32	23.7	65	98	148	37	25	38
nFCM	0.74	2.9	57	101	153	30	34	36
CLS	0.66	28.7^b^	–	87	133^c^	0	30	70^c^
AF4‐MALS	0.77	–	–	96	148	6	37	57

^a^ 3 peak Gaussian fit R^2 ^= 0.992.

^b^ only 2 repeat runs.

^c^ for both runs there are small satellite peaks (< 2%) at 108 and 165 nm respectively, which were ignored.

The three populations in sample D (100 nm + 150 nm + 200 nm) were resolved with NTA, TRPS, nFCM, CLS and AF4‐MALS, as shown in SI (Figure S5). Sample J, the most challenging trimodal mixture that contains the smaller particle population of 60 nm, can be fully resolved only by TRPS and nFCM. NTA, on the other hand, underestimates the proportion of the 60 nm particle in the mix J (∼2% instead of 33.3%). Repeat runs of sample J measured at identical instrumental settings but on different days (Figure S6, Table S6) showed that all three populations within the mix can be resolved (although not fully). Nevertheless, the 60 nm population was still underestimated, being 4%‐5%, instead of 33.3%. Total measured concentration values between original and repeated runs are comparable (see Table S6).

Similarly, NTA struggles to resolve CPN150 and CPN200 in sample D (SI/Figure S5). Therefore, in order to estimate the % distribution of the various modes within the mix and the average diameter of each population, the PSD was fitted with 3 Gaussians (see Figure S6) for both samples J and D.

CLS fails to detect the 60 nm particles within the mix but can clearly resolve the 100 nm and 150 nm populations. In fact, 50–60 nm is close to the smallest size limit, detectable when analysing polystyrene particles with CLS in sucrose gradient, and the actual concentration of the 60 nm particles within the mix is not sufficient to detect them with CLS. Moreover, CLS needs an involved post‐processing data analysis, especially when measuring low concentration samples and approaching the smallest detectable size limits. The CLS inset in Figure 2 exemplifies the need for baseline‐subtraction and prior knowledge of the composition of the sample for accurate CLS concentration measurement. The mass‐weighted PSD shows several distinguishable small peaks below 80 nm (Figure 2, CLS inset, blue curve, baseline‐subtraction in red; baseline has been omitted at a peak around 60 nm), most probably none of them being due to particulates in the sample but baseline drift. The baseline has been omitted at one of these peaks, situated approximately at 54 nm, which could be possibly caused by the 60 nm polystyrene particles, however the peak is too narrow to represent real particles. Measured maximum concentrations without baseline‐subtraction can be up to 50x higher (see sample G; Figure S1) than respective concentrations from integration of baseline‐subtracted PSDs.

AF4‐MALS also fails to detect the smallest 60 nm particles in the calculated number‐based PSD and underestimates the CPN100 versus CPN200 ratio, showing that in presence of multiple populations, despite the AF4 separation prior to detection, the higher noise/signal ratio associated to light scattering data of smaller particles can still partially bias the %wt evaluation calculated by applying the density plot algorithm.

Measured total concentrations are all within +/‐90% of the nominal concentration (see Table 6), with TRPS, baseline‐corrected CLS, AF4‐MALS and nFCM all being within ∼30% of the nominal concentration. When the populations are resolved, a numerical value for the mode and averaged diameters can be reported. Particle diameters associated to the various populations within the mixtures of samples D (see SI/Figure S4) and J are in good agreement with nominal diameters, for NTA, TRPS, nFCM and AF4‐MALS measurements, but not for the CLS measurement. As in case of the monomodal samples, the sizes measured by CLS are consistently smaller (by up to 11%) than nominal values, possibly due to an incorrect estimation of the true density of the nanoparticles.

#### 3.3.2 Quadrimodal mixtures

**FIGURE 3:**
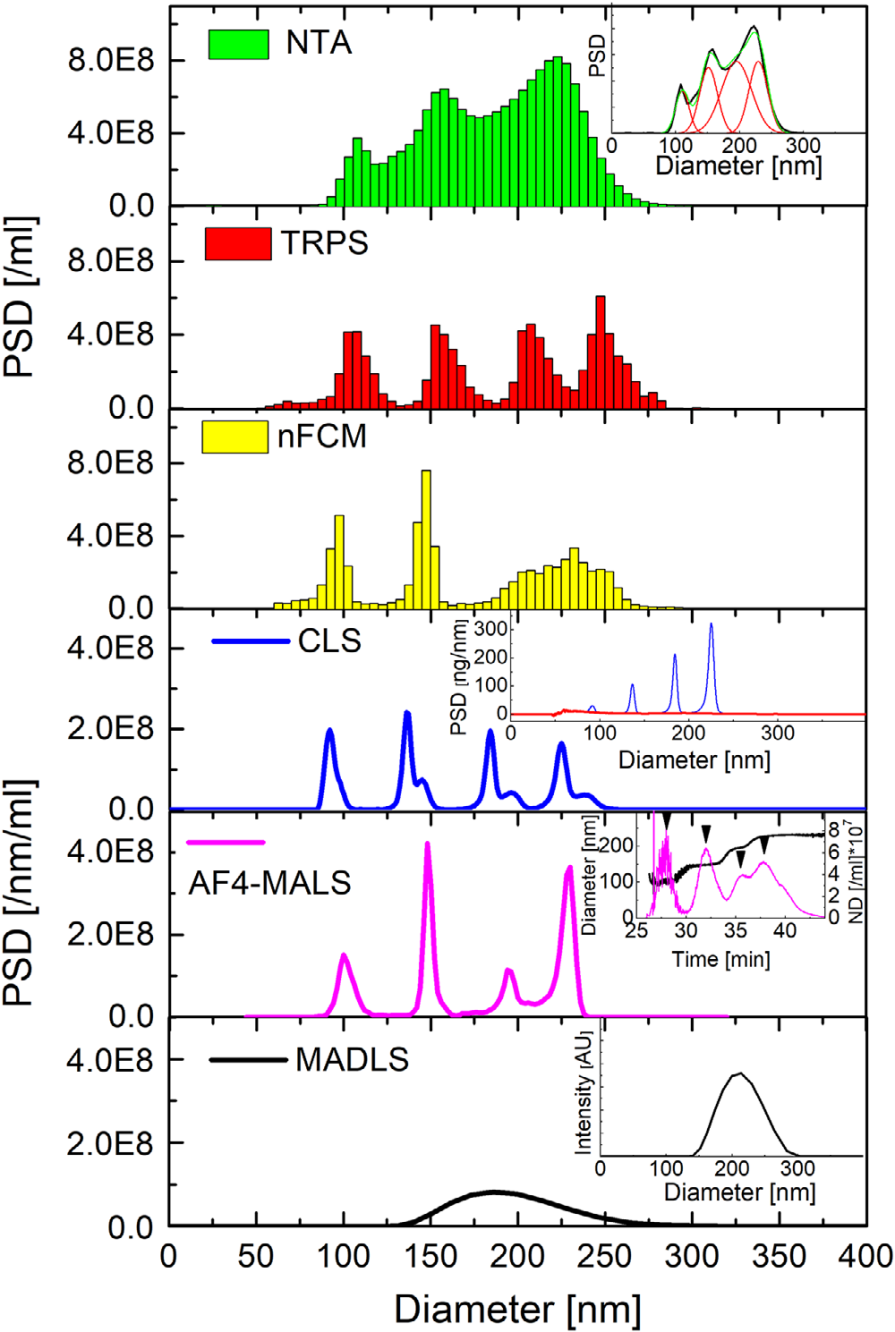
NTA, TRPS, CLS, nFCM, AF4‐MALS and MADLS measurements of quadrimodal sample C (CPN100/CPN150/CPN200/CPN240 at 25/25/25/25). NTA, TRPS, nFCM and MADLS measurements were averaged over 3 runs and CLS over 2. AF4‐MALS was only performed once. The NTA inset shows the multi‐Gaussian fit of its PSD. The CLS inset shows the base‐line correction of the respective mass‐weighted PSD, whilst the AF4‐MALS inset shows the number density‐based fractogram and sample specific particle peak positions. The MADLS inset shows the intensity‐weighted PSD

**FIGURE 4:**
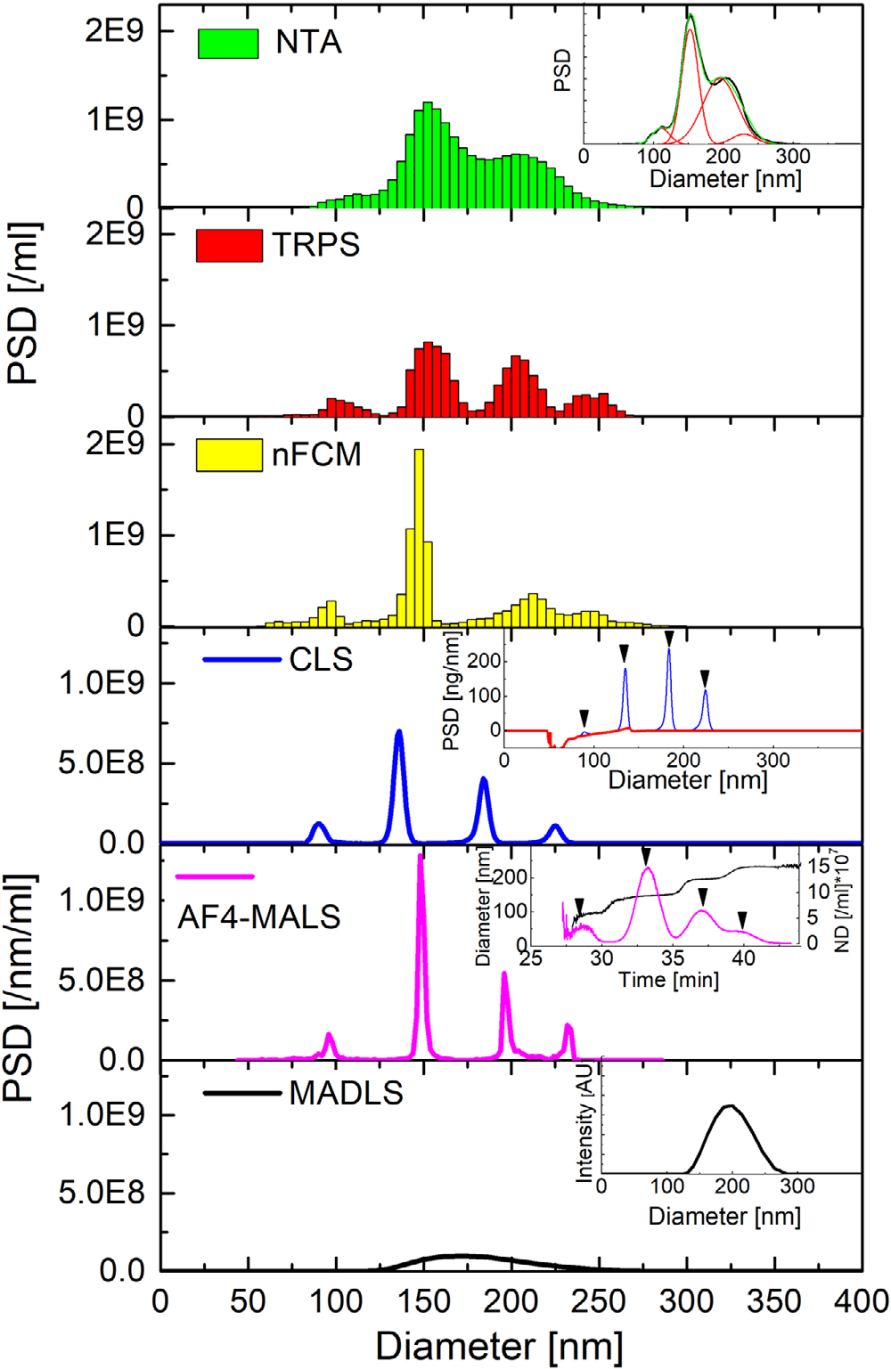
NTA, TRPS, nFCM, CLS, AF4‐MALS and MADLS measurements of quadrimodal sample I (CPN100/CPN150/CPN200/CPN240 at 10/50/30/10). NTA, TRPS, nFCM and MALDS measurements were averaged over 3 runs and CLS over 2. AF4‐MALS was only performed once. The NTA inset shows the multi‐Gaussian fit of its PSD. The CLS inset shows the base‐line correction of the respective mass‐weighted PSD, whilst the AF4‐MALS inset shows the number density‐based fractogram and sample specific particle peak positions. The MADLS inset shows the intensity‐weighted PSD

Quadrimodal mixtures of polystyrene were tested as the next step (samples C and I). CLS, AF4‐MALS, TRPS and nFCM can resolve all four populations within the mix for the quadrimodal samples C and I (Figures 3 and 4), whilst NTA cannot resolve the mix sufficiently. NTA repeat runs of sample C, measured at identical instrumental settings but on different days, showed some inter variability in the resolution power between NTA replicates (SI/Figure S6). These observations lead to further optimization of NTA measurements, that will be pursued in a follow up methodological study. MADLS only shows a broad distribution, and as for all multimodal samples it cannot resolve various modes within the mixtures. Again, CLS and NTA require post‐processing of the data, involving baseline‐subtraction for CLS (see insets to CLS in Figures 3 and 4) and fitting with 4 Gaussian distributions for NTA (see Figure 3 and 4, NTA insets) in order to calculate %distributions of various populations/modes within a mix (Tables 7 and 8).

**TABLE 7:** Measured mean concentrations (including CVs), mode diameters for various populations within the mix and respective % distributions for quadrimodal sample C (CPN100/CPN150/CPN200/CPN240 at 25/25/25/25)

Technique	Mean concentration (NPs/ml)*10^10^	CV (%)	Diameter (nm)100/152/203/240				% Distribution25/25/25/25			
NTA	1.58	9.8	106^a^	156^a^	215^a^	–	7^a^	40^a^	53^a^	–
			*110* ^b^	*151* ^b^	*155* ^b^	*230* ^b^	*11* ^b^	*22* ^b^	*41* ^b^	*26* ^b^
TRPS	0.85	25.5	105	153	208	248	23	22	24	31
nFCM	0.66	11.7	98	147	203	231	20	28	22	25
CLS	0.74 *0.87* ^c^	47.9^d^ *19.7* ^c^	94	141	191	232^e^	23 *37* ^c^	28 *25* ^c^	24 *19* ^c^	25 *19* ^c^
AF4‐MALS	1.00		100	148	195	230	17	29	14	40
MADLS	0.63	13.6	214 *187* ^f^	–						

^a^ 3‐peak Gaussian fit R^2 ^= 0.994.

^b^ 4‐peak Gaussian fit R^2 ^= 0.998 (slanted); fixed peak positions for 200 (195.07) and 240 (229.42) nm modes.

^c^ slanted values are derived without baseline‐subtraction.

^d^ only 2 repeat runs.

^e^ there are two small satellite peaks (< 2%) at 166 nm and 224 nm for run 2, which were ignored.

^f^as derived from PCD (slanted) as opposed to intensity‐weighted PSD.

**TABLE 8:** Measured mean concentrations (including CVs), mode diameters for various populations within the mix and respective % distributions for quadrimodal sample I (CPN100/CPN150/CPN200/CPN240 at 10/50/30/10)

Technique	Mean Concentration (NPs/ml) *10^10^	CV (%)	Diameter (nm)100/152/203/240				% Distribution10/50/30/10			
NTA	1.56	6.5	110^a^	153^a^	201^a^	–	5	46	49	–
			*111* ^b^	*152* ^b^	*195* ^b^	*229* ^b^	*6* ^b^	*41* ^b^	*48* ^b^	*5* ^b^
TRPS	0.99	4.9	98	153	203	248	10	44	33	13
nFCM	0.87	3.1	98	147	213	247	10	52	25	10
CLS	1.02 *1.07* ^c^	0.5^d^ *20.4* ^c^	90	136	184	225	10 *8* ^c^	50 *52* ^c^	30 *30* ^c^	10 *10* ^c^
AF4‐MALS	1.14	–	96	148	196	232	12	52	26	10
MADLS	0.73	6.3	195 *173* ^e^	–						

^a^ without knowing the composition the distribution would suggest a 3‐peak fit (R^2 ^= 0.998).

^b^ 4 peak Gaussian fit R^2 ^= 0.997 (slanted); fixed peak positions for 200 (195.07) and 240 (229.42) nm modes for the 4‐peak fit.

^c^ no baseline‐subtraction (slanted).

^d^ only 2 repeat runs.

^e^ as derived from PCD (slanted), as opposed to intensity‐weighted PSD.

Figure 3 shows the measured PSDs for 1:1:1:1 mix of CPN100, CPN150, CPN200, and CPN240. Mean diameters of various populations within the mix are in good agreement with nominal diameters (within 10%) for all techniques (see Table 6), with CLS again showing consistently lower diameters (by up to 11%). NTA shows only 3 peaks at 108nm, 158 nm and 223 nm, with best 3‐peak Gaussian fit centred around 106 nm, 156 nm and 215 nm respectively (see Table 6). For the fitting of the NTA PSD with 4 Gaussians, the diameters of the CPN200 and CPN240 Gaussians had to be fixed to 195 nm and 229 nm, in order to get realistic fits. These were the peak positions of the respective monomodals as measured with NTA.

In terms of analysing PSDs, NTA measurements required multimodal fitting, whilst TRPS, CLS and AF4‐MALS can resolve the four populations within the mix without need for post processing. Repeat measurements of nFCM are slightly size‐shifted and hence when averaging the 3 repeat runs, resolution is lost, and the 200 nm and 240 nm peaks cannot be resolved anymore. However, for single nFCM measurements these peaks can still be resolved (see SI/Figure S7). CLS shows a double peak structure due to the size shift between repeat runs, again showing some repeatability issues (Figure 3 and Figure S7). For AF4‐MALS, number density‐based PSDs derived with the custom‐made Macro provided by Wyatt is able to resolve all four populations within the mix.

MADLS again shows a very broad distribution, centred around 195 nm without any features, that would indicate a multimodal composition. In the MADLS inset the respective intensity‐weighted PSD is shown with a mode diameter of 214 nm.

In terms of %distribution of various modes in the mix, nFCM, TRPS, and CLS (the latter after baseline‐subtraction) can satisfactorily measure the nominal 1:1:1:1 ratio (CV 3%), whilst for NTA the highest represented population within the mix can be up to ∼4 times higher than the lowest represented population (see Table 7). Measured total concentrations as measured with NTA, TRPS, nFCM, CLS and AF4‐MALS are all within +/‐60% of the nominal concentration. Only MADLS deviates a bit more from the nominal concentration. AF4‐MALS significantly underestimated the proportion of CPN100 and CPN200 within the mix.

The measured PSDs for 10:50:30:10 mix of CPN100, CPN150, CPN200, and CPN240 are shown in Figure 4. Mean diameters of various populations within the mix are in good agreement with nominal diameters (within 12%) for all techniques, with CLS again showing consistently lower diameters than the other techniques. NTA shows only 3 peaks at 103 nm, 153 nm, and 203 nm respectively (see Table 8), whilst nFCM, TRPS, CLS and AF4‐MALS can resolve all four populations within the mix. For the fitting of the NTA PSD with 4 Gaussians, the diameters of the CPN200 and CPN240 Gaussians had to be restrained to 195 nm and 229 nm, in order to get realistic fits. These were the peak positions of the respective monomodal CPN 200 and CPN240, as measured with NTA.

Again, baseline‐subtraction of CLS PSDs is crucial to analyse the concentration and %distribution. In this specific case the mass‐based PSD, as measured with CLS (Figure 4, CLS inset, blue curve) shows negative values for diameters smaller than ∼120 nm. These negative values then translate into negative particle numbers which obviously have no physical meaning. Only baseline‐subtraction (Figure 4 CLS inset, blue curve) transforms datasets into a format that allows the analysis of mass‐ and number‐weighted PSDs.

In terms of %distribution of various modes in the mix TRPS, nFCM, CLS (after baseline‐subtraction), and AF4‐MALS can measure the nominal 10/50/30/10 ratio satisfactorily (CV 3%) (see Table 8). NTA overestimates the proportion of CPN200 and there needs to be a knowledge of the composition at hand in order to predict the 240 nm particles within the mix (see Table 8). As for all other multimodal samples MADLS totally fails to resolve the particle populations within the mix. Measured total concentrations as measured with NTA, TRPS, nFCM, CLS and AF4‐MALS are all within +/‐60% of the nominal concentration.

### 3.4 Concentration measurements summary

To summarize, there is a very good agreement (within 90%) of measured polystyrene sample concentrations between the six different techniques as reported in Table 9, especially if the outliers of sample G for NTA and sample H for AF4‐MALS are excluded. The only marginal outlier is NTA, that overestimates the nominal concentration (Table 9). This overestimation of concentration by NTA is in line with recent results reported by Bachurski et al. (2019) and Vestad et al. (2017), where the concentration of polystyrene and silica nanoparticle standards (100‐150 nm) was overestimated by approximately 100% and 130%‐160% for Nanosight NS300 and NS500 respectively. Possible reasons for this overestimation are related to the choice of measurement parameters (camera settings and detector threshold) which are optimized during the calibration steps with the indicated metrology standards. A more sophisticated approach for fine‐tuning the NTA measurement, avoiding the operator subjective setup, at different size ranges is under development and will be matter for a follow up paper. Furthermore, the use of a hyphenated technique such as FFF coupled with NTA has recently been presented by Drexel et al. (2020). Also, the adoption of short wavelength lasers and suitable standard controls will allow for more reliable measurements, in particular of small biological nanoparticles with low refractive index.

**TABLE 9:** Summary of measured polystyrene sample concentrations with various techniques

Sample	NTA*10^10^/ml	TRPS*10^10^/ml	nFCM*10^10^/ml	CLS^a^*10^10^/ml	AF4‐MALS*10^10^/ml	MADLS*10^10^/ml
A	1.32	1.00	–	1.39	0.84	0.55
B	1.39	1.10	0.94	1.14	1.01	1.20
C	1.58	0.85	0.66	0.74	1.00	0.63
D	1.33	0.95	0.66	0.82	0.84	0.65
E	1.85	1.15	0.72	1.08	0.97	0.85
F	1.10	1.06	0.79	0.77	1.07	1.43
G^b^	4.31	1.18	–	1.04	1.09	0.77
H	2.06	1.33	0.96	1.11	5.80	0.62
I	1.56	0.99	0.87	1.02	1.14	0.73
J	1.88	1.32	0.74	0.66	0.77	–
Mean	1.84 (1.56^c^)	1.09	0.79	0.98	1.45 (0.97^d^)	0.83
CV [%]	50 (20^c^)	14	15	23	105 (13^d^)	36

*baseline‐corrected PSDs.

^b^ concentration of sample G with a nominal concentration of 10^9^/ml was multiplied by 10 to be comparable with all other samples at a nominal concentration of 10^10^/ml.

^c^ NTA results, excluding the outlier of sample G.

^d^ AF4‐MALS results, excluding the outlier of sample H.

### 3.5 Measurement of liposome samples

For light scattering based techniques liposomes could be considered as another reference material (Simonsen, 2016; Valkonen et al., 2017) for EVs with more similar optical properties than polystyrene standards. However, differently from NIST traceable standards that are recognized reference material for size measurements, no standard liposomal formulations exist yet, either for size, nor for concentration measurements. For this reason, the authors decided to develop the protocols with NIST traceable polystyrene standards and then to validate them with liposomes before measuring the EV isolates. In this context, a commercially available and well characterized (certificate of analysis provided) PEGylated liposome sample (containing three different lipids: Cholesterol, HSPC and MPEG‐DSPE) was chosen, that is very monodisperse with a mean nominal size of 78 nm.

Despite not reported in the certificate of analysis, the number‐based concentration of liposomes (/ml) can be calculated as described below. Theoretical calculations (Epstein et al., 2006; Szoka & Papahadjopoulos, 1980) for approximating the number‐based concentration of liposomal preparations assume an average size of spherical particles, that has been determined with a suitable nano‐sizing instrument, such as the instruments/techniques discussed in this study. The assumption of all liposomes having the same size is acceptable, if the formulation is quite monodisperse, which is the case for the commercial liposome formulation, pertinent to this study. In addition, it is difficult to accurately assess the surface area and volume of the phases in liposomal formulations due to the multiple or single double‐layered membranes, and the three‐dimensional arrangement of the various lipids (Epstein et al., 2006). We uses a surface area method as described in (Epstein et al., 2006), to determine the number concentration of liposomes. The number of liposomes can be expressed as indicated in equation 1:

(1) $$N=\frac{S_{tot}}{S_{layer}*\left(number\;of\;lipid\;layers\right)},$$

with *S_tot_* being the surface area of all the vesicles in the suspension based on the area occupied by all lipid molecules, and S_layer_ is the mean surface area of one layer in one vesicle based on its mean radius (in our case 78 nm). *S_tot_* can be estimated as from equation 2:

(2) $$S_{tot}=N_{A}*(n_{Cholesterol}*S_{Cholesterol}+n_{HSPC}*S_{HSPC}+n_{MPEG-DSPE}*S_{MPEG-DSPE},$$

Where *N_a_* is the Avogadro constant, *n* the number of moles for each lipid type in the suspension and *S* the respective packing area per lipid head group. We assumed packing areas of 0.28 nm^2^, 0.7 nm^2^ and 0.5 nm^2^ for Cholesterol, HSPC and MPEG‐DSPE respectively (Dickey & Faller, 2008; Epstein et al., 2006; Mullerlandau & Cadenhead, 1979; Small, 1967). Assuming a bilayer structure, and deriving the number of moles from the mass ratio of lipids, molar mass and total lipid weight per ml, the total concentration of liposomes was calculated to be 2.14 * 10^14^/ml.

**FIGURE 5:**
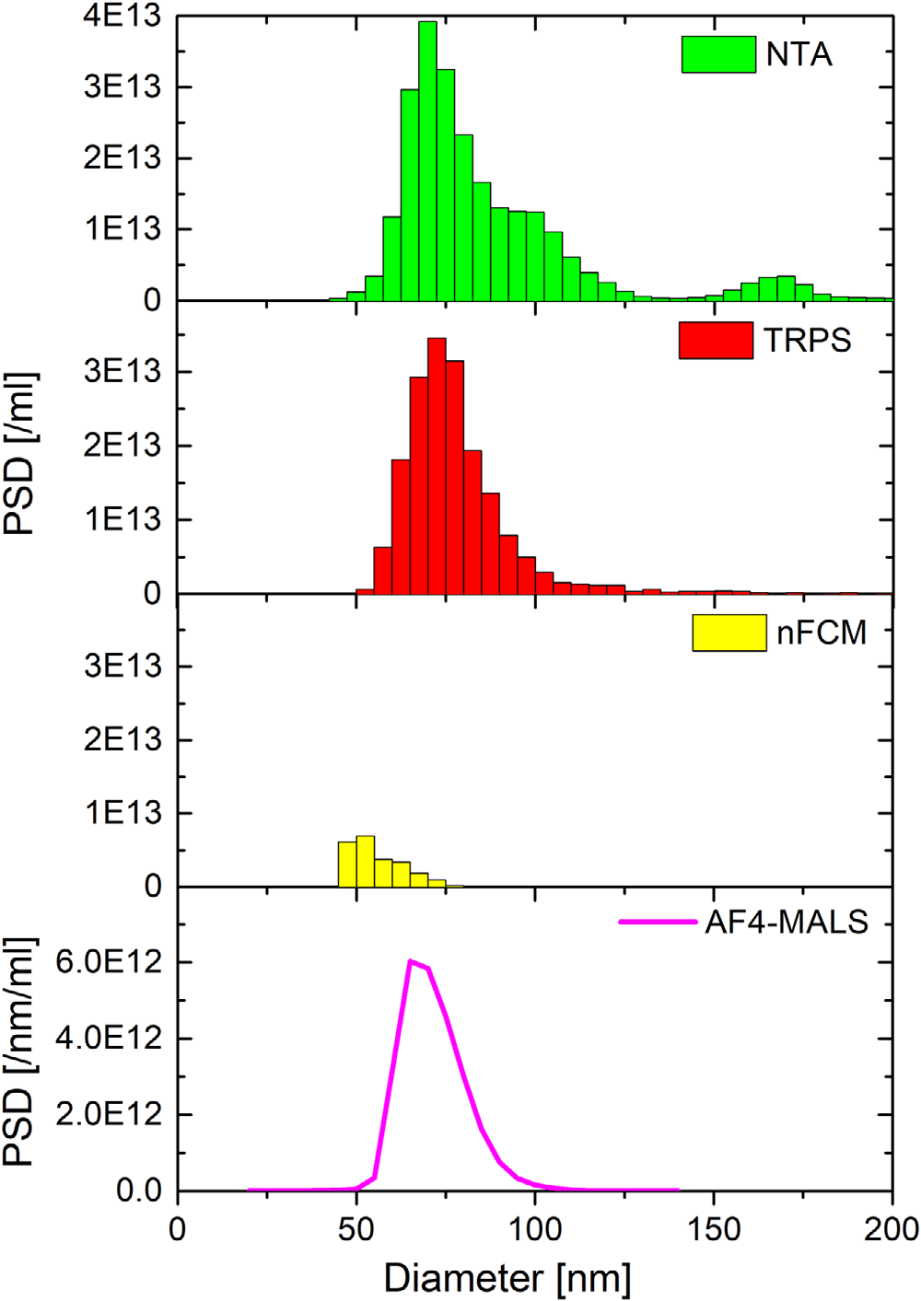
Number‐weighted PSDs of PEGylated liposome sample, as measured with NTA, TRPS, nFCM and AF4‐MALS. All results are averaged over 3 repeat runs each

The calculated liposome number concentration is in good agreement with the mean concentration (1.87*10^14^/ml), averaged over concentration measurements, taken with NTA, TRPS, and AF4‐MALS (see Table 10 and Figure 5). Whilst NTA, TRPS and AF4‐MALS concentrations agreed well (CV = 27%), nFCM measured a significantly lower concentration than all other methods (up to 11 times lower than other techniques). The coefficient of variance of the concentration results from all for techniques is 47%, with nFCM measuring by far the lowest, and NTA measuring the highest concentration. In terms of the liposome diameter, NTA, TRPS and AF4‐MALS are within 10% nm of the nominal average liposome size, whilst the nFCM PSD is significantly skewed to smaller diameters, with a mode diameter of 53 nm. This shift of the PSD towards lower diameters for nFCM could be due to the use of a silica nanoparticle size calibration with a higher refractive index than the solid particle equivalent refractive index of the liposomes.

**TABLE 10:** Mean concentration, CV and mode diameter of liposome sample, as determined with NTA, TRPS, nFCM and AF4

Technique Used	Mean concentration (NPs/ml)*10^14^/ml	CV (%)	Mode diameter [nm]
NTA	2.4	16.3	70
TRPS	1.8	9.5	73
nFCM	0.23	3.0	52
AF4‐MALS	1.4	2.2	65
CLS	–	–	–

Interestingly, the AF4‐MALS PSD concentration results are very sensitive to refractive index changes. Determining the refractive index of non‐uniform biological nanoparticles is not straightforward and hence concentration results might have a high uncertainty, due to the ill‐defined refractive index. For example, when estimating the refractive index of the liposomes as 1.45 instead of 1.392 (Matsuzaki et al., 2000) as currently done, the measured liposome concentration will significantly decrease from 1.3*10^14^ ml to 1.6*10^13^/ml.

The liposome sample could not be measured by CLS, due to the small size and to the small difference in density between the particles and the sucrose gradient used in the measurements (data not shown). For liposome measurements with centrifugal techniques analytical ultracentrifugation could be considered, where higher centrifugal force can be applied and particles are measured in their own buffer as reported by (Mehn et al., 2017). However, since AUC was not included in this study, the measurement was not repeated here, and will be the subject of a new study.

### 3.6 Measurement of EV containing plasma samples

Finally, in the progression from the measurement of monomodal, to multimodal polystyrene and liposomes, we report the measurement of EV containing plasma samples (as complex biological sample) by NTA, TRPS, nFCM, CLS and AF4‐MALS. MADLS measurements of EV isolates were not attempted, because based on results of polystyrene standard EV mimics and the polydisperse nature of EV isolates, MADLS was not able to resolve the PSD of a polydisperse sample in the size range of the EV isolates.

**FIGURE 6:**
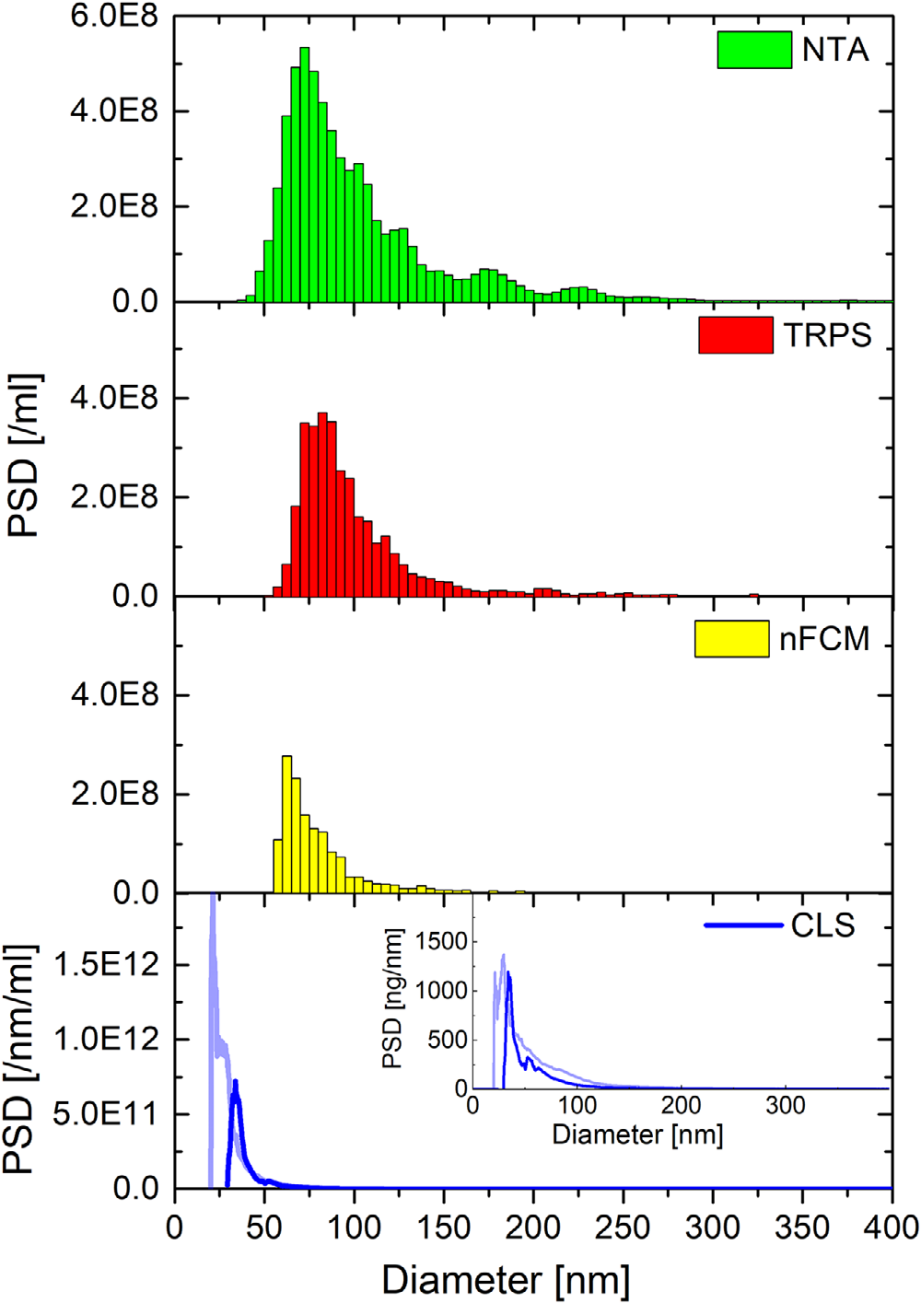
Number‐weighted PSDs of EV containing plasma sample, as measured with NTA, TRPS, nFCM and CLS. NTA and TRPS results are averaged over 3 repeat runs each. For CLS the two repeat runs are shown in order to demonstrate the discrepancy between repeat runs. Respective mass‐weighted PSDs are shown In the CLS inset. nFCM was only performed once

There is quite a big difference between EV isolate concentrations as measured with different techniques. To make results comparable across various techniques, concentrations were evaluated over the size ranges 80‐250 nm and 50‐400 nm. Whilst nFCM, NTA and TRPS concentration results are of the same order of magnitude, the CLS results are two orders of magnitude higher (Figure 6, Table 11). Of the three single particle analysis techniques NTA measured the highest EV concentration, whilst nFCM measured the lowest. NTA and TRPS were averaged over three runs with CVs being 19% and 1% respectively, whilst nFCM measurement was only performed once.

**TABLE 11:** Mean concentration, CV and mode diameter of EV containing plasma sample, as determined with NTA, TRPS, nFCM and CLS

Technique Used	Mean concentration (NPs/ml)	CV (%)	Mode diameter [nm]
NTA	C_50‐400 _= 5.9*10^9^ C_80‐250 _= 3.5*10^9^	19.4	82
TRPS	C_50‐400 _= 3.3*10^9^ C_80‐250 _= 2.9*10^9^	1.1 1.9	80
nFCM	C_50‐400 _= 1.4*10^9^ C_80‐250 _= 5.2*10^8^	N.A.^a^	63
CLS	C_50‐400 _= 7.355*10^11^ C_80‐250 _= 9.364*10^10^	6.4 33.5	28

^a^ Only one repeat.

The two repeat runs of number and mass‐weighted PSDs for CLS are also shown in Figure 6, however the total concentrations are much higher than determined by nFCM, NTA and TRPS and most probably due to the noise in the primary data that leads to severe artefacts in the PSD. As previously discussed, for CLS the sample composition and size range need to be well known and baseline‐subtraction undertaken, in order to calculate the concentration of particles in the sample. In case of the EV isolate sample or any polydisperse sample with similar properties, the required data post‐processing (baseline subtraction) for CLS becomes impossible, and hence measured concentrations will have a very high uncertainty.

AF4‐MALS analysis is not reported since we failed to obtain a sample recovery > 70%, as required by ISO TS 21632, when analysing the EV containing plasma samples (data not shown), measured in two different laboratories on the same EV sample, although in both cases the measurement protocol was in agreement with a recently published protocol that should enabled the measurement of EV isolates with AF4‐MALS (Zhang & Lyden, 2019). Under the assumption that recovery in the results provided by Zhang & Lyden, (2019) was checked and > 70%, as required by the ISO documents, our lower recovery may be due to the relatively low concentration of our EV isolate sample, or due to a specific absorption of the sample measured in this study on the semipermeable membrane. As follow up of this study additional measurements of the EV isolate samples should be performed to better understand the discrepancy between these results. AF4‐MALS could also considered the technique of choice to fractionate small lipoprotein fractions from EV vesicles and to analyse batch to batch consistency during purification processes. This is under evaluation in another study.

### 3.7 Comparison of the different platforms for particle concentration measurements

The performance of the six techniques regarding to measured concentration and number‐weighted PSD, are summarized in Tables 1 and 9. The results obtained in the previous section for the measured polystyrene samples, liposomes and of EVs, have underlined some method specific issues that we would like to discuss more in detail in this section, in a method by method technical approach.

In the analysis of polystyrene mixtures, **NTA** was able to adequately resolve bimodal populations of CPN100 and CPN200 but showed limitations to properly resolve trimodal and quadrimodal samples. Similar observations were made in (Anderson et al., 2013). Whilst monomodals of CPN100 and CPN150 and CPN200 show distinct and separate distributions (see Figure 1), they do overlap when mixed in an equal ratio (samples C, D and J). Measured hydrodynamic diameters agree very well with nominal mode diameters (within 5%) for all samples, where the population within the mix could be clearly resolved. Multimodal Gaussian fits were used to determine % distributions. In cases of tri‐ and quadrimodal samples C, I and J multi‐Gaussian fittings were non‐obvious and prior knowledge of the exact composition of the mixtures was required, in order to calculate % distributions reliably. For all samples but sample G, measured concentrations are within two‐fold of the nominal concentration. NTA slightly overestimated sample concentrations when measuring polystyrene mixtures. The total mean concentration and average CV of all sample A‐J (with sample G concentration multiplied by 10) are 1.56*10^10^/ml and ∼20% respectively, when the outlier of sample G was removed (Table 9). Please also note that, according to previous studies there can be variability in the concentration and size distribution measured of a reference material between differing NTA platforms (Valkonen et al., 2017). Repeatability, reproducibility, and the particle concentration overestimation issues observed in this work may be linked to the subjective choice of some measurement setting, that must be carefully fine‐tuned, depending on the particle size and on their optical properties to be resolved. An approach to reduce these issues is currently under investigation by some of the authors.

**TRPS** resolved various populations of polystyrene particles (up to 4) within each sample very well, which is in agreement with a study by Anderson et al. (Anderson et al., 2013). In the analysis of the polystyrene samples, the calculated mode diameters were in good agreement (within 5%) with the nominal diameters of the various NIST‐traceable standards. Concentration ratios of various populations within a multimodal sample, as measured with TRPS accurately represent the experimental mixing ratios. The total mean concentration and average CV of all sample A‐J (with sample G concentration multiplied by 10) are 1.07*10^10^/ml and ∼14%, respectively (Table 9). The reproducibility of the measured concentrations is very good, with most triplicate concentration measurements lying within 10% for polystyrene mixtures and 2% for EV isolates.

**nFCM** resolution of monomodal and four‐modal polystyrene particles was very good, consistently calculating mode diameters in strong agreement with nominal diameters (4%). Standard procedure is to provide histogram bins of 0.5 nm but were adjusted to fit alongside other methods in Figures 2,3,4, demonstrating very narrow PSDs for sub populations. Concentration ratios of multimodal samples J and I proved to be accurate, with only minor discrepancies occurring for sample C. Repeat runs for sample C were slightly size shifted and in the process of averaging 3 repeat runs resolution was lost. Total particle concentrations for each sample were consistently accurate, except for sample E which showed high particle contamination (< 60 nm) which was removed from data analysis by thresholding. The total mean concentration of all samples is 0.79*10^10^/ml and CV ∼15% (Table 9).

In the case of the measurement of polystyrene mixtures, **CLS** can resolve various particle populations within most multimodal samples, however in most cases a precise knowledge of the particle size within the mix is necessary to get reproducible results for %distributions and total concentrations. CLS failed to measure the 60 nm polystyrene particles at the applied concentrations within the 60 + 100 + 150 nm mix. Modal Stokes diameters of respective populations are consistently smaller (by up to 11%) than nominal hydrodynamic diameters, possibly due to an incorrect estimation of the apparent density of the polystyrene nanoparticles. Post‐processing of the measured mass‐ and number‐weighted PSDs is crucial to reliably predict and measure particle concentrations with CLS. Provided, PSDs are baseline‐subtracted and the composition of the sample is well known, the measured concentrations averaged over all 10 polystyrene samples are in perfect agreement (within ∼2%) with the nominal concentration of 10^10^/ml. The fact that there is a clear need for the knowledge of the particle density of a sample and data post‐processing, PSD and concentration measurements of real‐life samples using CLS will need to use independent estimations of the particle density. In the case of the EV isolate measurements, the total concentration measured by CLS was much higher than determined by NTA and TRPS, and most probably due to the relative high noise in the primary data that leads to severe artefacts in the PSD.

**AF4‐MALS** is well suited to measure the size of polydisperse samples, and successfully performed the measurement of particle concentration if size was > 60 nm (diameter). Importantly, for the first time, an approach to report PCD in particles/nm/ml was developed as outcome of this study, in a joint collaboration with Wyatt Technology. As demonstrated in this study, AF4‐MALS can resolve various populations within the polystyrene samples very well (with the exception of CPN60 of sample J), predicting mode diameters typically within 2% of nominal diameters and measure total particle concentration rather precisely both in case of polystyrene mixes and of PEGylated liposomes. However, the reader should be aware that the use of MALS detector has some limitations for concentration measurements, differently from online ICP‐MS and from sp‐ICP‐MS used for certain inorganic samples (Aznar et al., 2017; Cascio et al., 2015). There are two main reasons for this. First, the uncertainty of predicting the optical properties of unknown samples (knowledge of the refractive index of the particles) may crucially affect AF4‐MALS concentration measurements, that are derived by applying the Mie Theory on the light scattering data. Moreover, the sample can be lost in the measurement process, for example, due to the particle interactions with the membrane, that could bias the measurement of particle concentration if sample recovery is less than 70%‐80%. The authors cannot explain why the nature protocol reported by Zhang et al. was not successful in the analysis of the EV containing plasma sample producing a not acceptable sample recovery (< 70%) for particle sizing and concentration measurements. Possibly, the concentration of the EV containing plasma sample tested was too low in our study. It is also possible that the sample recovery of the EV containing plasma sample was very low due to the interaction of the vesicles with the membrane, possibly showing reproducibility issues of AF4‐MALS in the measurement of EVs, that should be further verified by larger inter‐laboratory studies.

Whilst **MADLS** predicted the mode diameters of the monomodal polystyrene populations very well (within 6%), it was not able to resolve the separate populations in any of the sample mixtures. PSDs of monomodal samples were too broad in order to allow the resolution of the various particle populations within the mix. The reported size resolution limit for this method is 2:1 for particles larger than 150 nm (e.g. CPN150 and CPN300) (Technical Note, 2018b). For smaller particles than 150 nm the angular dependence of the light scattering profile is lowered and results in a reduced resolution (Technical Note, 2018a). This might be a decisive factor why bimodal samples A and G of CPN100 and CPN200 cannot be resolved with MADLS. The sample concentrations, as measured with MADLS appear to be on average approximately 20% lower than nominal concentrations. Due to the failure in resolving the polystyrene mixtures it was not selected for the EV isolates analysis.

### 3.8 Conclusions and future perspectives

The scope of the study was to provide a comparative assessment for measuring particle concentration with six techniques (e.g., ensembled, hyphenated and single‐particle analysis) for nanoparticles and biological samples, and to challenge these across a four step measurement process with increasing complexity. Importantly, to exclude any subjective biases, measurements of monomodal and multimodal polystyrene samples were done in a blind fashion, with the instrument operators not knowing the content of the sample. An in‐depth analysis of the particle concentration and size distribution measurements has been presented. All six techniques have technical challenges which have been discussed in light of the nature of the measured samples.

By analysing the 10 polystyrene standard samples, ranging from 50‐300 nm, we have demonstrated that number‐weighted PSDs can be derived for all methods, and that there is good agreement in sample concentration (within 90%) between different platforms. However, in some cases, it was necessary to apply advanced data post‐processing to calculate particle concentrations reliably and reproducibly, such as manual baseline subtraction for CLS, or the use of a novel PSD calculation algorithm developed for AF4‐MALS. Interestingly, when a small population introduced in a multimodal sample, for example, 60 nm particles in trimodal sample J, some techniques starts to provide less accurate results, for example, CLS and AF4‐MALS could not detect the smaller population at the applied concentration. Finally, in terms of resolving power of complex mixes nFCM, TRPS, CLS and AF4‐MALS perform very well, whilst MADLS cannot resolve any of the multimodal mixes, and NTA requires post‐processing fitting in order to estimate % distributions of various overlapping populations within a mix.

The results obtained by measuring PEGylated liposomes, as a second step, have allowed to validate the approach previously developed on all the platforms tested. Concentrations and size distributions, as measured with NTA, TRPS, and AF4‐MALS were in good agreement (with a coefficient of variance of 27%), whereas nFCM measured a significantly lower concentration than all other methods (up to 11 times lower than other techniques). The mean concentration, averaged over NTA, TRPS and AF‐MALS was in excellent agreement (within 15%) with the calculated liposome number concentration, as derived from lipid packing areas, molar ratios of lipids in the formulation, and the mean diameter of the composite liposomes (see Table 10 and Figure 5).

The measurement of extracellular vesicles as complex biological fluid has been the most challenging. Concentrations and size distributions (in the size range from 80–250 nm) of plasma EVs were well within one order of magnitude for NTA, TRPS and nFCM measurements. This shows that single particle analysis techniques are well suited to measure particle concentration of biological samples such as EVs. For CLS the measured concentration was significantly higher compared with the single particle analysis techniques, and for AF4‐MALS the concentration was below the acceptable threshold for size and concentration measurements, showing some limitations that have to be further investigated.

To conclude, this work is well aligned with the extensive effort undertaken by the EV community started in 2014, to develop reproducible methodologies for the harmonization of particle concentration and size distribution measurements. With the view to provide highly reproducible and repeatable data, we suggest considering multiple orthogonal approaches, with at least one hyphenated or non‐optical technique. The aim is to achieve maximum representative characterization and data robustness. This represents an initial step on the identification and quantification of the critical attributes in the characterization of EVs. Reproducible and accurate phenotyping and quantitative evaluation of other plasma components such as intermediate‐density lipoprotein, low‐density lipoprotein, and high‐density lipoprotein is also critically needed as part of the accurate EV characterization for the regulatory approval and full potential industrial exploitation.

## CONFLICT OF INTEREST STATEMENT

R.V. is a contractor at IZON Science, J.M. and M.M. are employed by IZON Science and their contributions to this paper were made as part of their contract/employment.

A.L., B.P., D.A. are employees of NanoFCM and their contributions to this paper were made as part of their employment.

## Supporting information

Supplementary information

## APPENDIX A GLOSSARY

*Diameter of gyration (Dg) *= is the root mean square distance of the object's parts from its centre of mass, as measured for example by MALS.

*Geometric diameter* = equivalent sphere diameter as measured with TRPS.

*Hydrodynamic diameter (Dh) *= Diameter of a sphere that possess the same diffusion coefficient (derived by the Stokes‐Einstein equation) as the particles in the applied conditions. It is indicative of the apparent size of the hydrated/solvated particles.

*Mass‐based particle concentration *= mass of particles/ml measured in a sample. It could refer to the total mass of particles/ml, if integrated over the whole mass‐weighted particle size distribution or to the particle mass, associated to single populations of particles which compose a polydisperse sample.

*Number‐weighted particle concentration *= number of particles/ml measured in a sample. It could refer to the total number of particles/ml, if integrated over the whole number‐weighted particle size distribution (total particle concentration) or to the particle concentration associated to single populations of particles which compose a polydisperse sample.

*Particle concentration distribution (PCD) *= number‐weighted particle size distribution where the relative amount is expressed as particle number per ml and bin size in nm, and not in arbitrary or normalised units versus size.

*Particle size distribution (PSD) *= mathematical function that defines the relative amount, typically by particle mass or particle number, of particles present according to size.

*Nominal distribution width = *2xstandard deviation of a monodispersed particle distribution, as determined with transmission electron microscopy by the particle provider.

*Single particle analysis *= analytical methods able to detect and counts single particle events.
